# Generative real-time planning for multi-robot contingencies using deep prior-guided whale optimization

**DOI:** 10.3389/frobt.2026.1955600

**Published:** 2026-09-18

**Authors:** Xinyi Zhang, Xinqi Li, Wenbo Li

**Affiliations:** Xi’an High-Tech Research Institute, Xi’an, China

**Keywords:** contingency management, deep prior learning, guided whale optimization algorithm, mission planning, multi-robot coordination

## Abstract

**Introduction:**

Dynamic multi-robot coordination demands real-time resilience against stochastic disruptions, yet existing planning methodologies often falter under the computational burden of high-dimensional state transitions. To address this challenge, we present a generative real-time mission planning framework that integrates deep prior learning with adaptive evolutionary optimization.

**Methods:**

By employing a subtraction-average-based optimizer (SABO) to tune long short-term memory (LSTM) networks, we extract deep spatiotemporal priors that map discrete contingency events to continuous cost intervals, effectively compressing the feasible search space. Within this refined subspace, a guided whale optimization algorithm (GWOA), which is augmented by target-gap adaptive control and dimension-wise opposition learning, executes precise plan regeneration.

**Results:**

Validation across six distinct contingency scenarios, ranging from risk-induced termination to agent paralysis, demonstrates that the proposed approach outperforms traditional reallocation baselines, achieving rapid responsiveness and superior solution stability. The framework also demonstrates scalability in large-scale scenarios involving up to 1000 agents.

**Discussion:**

These results demonstrate that integrating deep spatiotemporal prior learning with adaptive evolutionary optimization can effectively reduce the computational burden of mission replanning while maintaining responsiveness, stability, and scalability, providing a robust approach to resilient multi-robot management in unpredictable environments.

## 1 Introduction

Multi-robot systems have emerged as the cornerstone of modern automation, orchestrating complex logistics, disaster response, and planetary exploration missions with unprecedented efficiency (Yoshida et al., 2025; Sathyan et al., 2020; Kwa et al., 2022; El Romeh and Mirjalili, 2023). By distributing tasks across heterogeneous agents, these systems can achieve capabilities far exceeding the sum of their individual components. However, the transition from controlled, static environments to stochastic real-world scenarios introduces uncertainty, which is a critical operational bottleneck (Lacerda et al., 2022). In dynamic environments, mission execution is frequently disrupted by unforeseen contingencies, ranging from sudden agent paralysis and fluctuating communication bandwidth to the abrupt injection of high-priority tasks. Consequently, the ability to reconfigure mission plans in real-time, ensuring both feasibility and optimality while mitigating system-wide perturbations, has become the key problem in resilient autonomy.

The fundamental challenge lies in the computational intractability of dynamic reallocation (Lin et al., 2022). Multi-robot task allocation under complex spatiotemporal constraints is intrinsically an NP-hard problem (Bounaira et al., 2024). Traditional exact solvers, while precise, suffer from exponential time complexity, rendering them prohibitive for real-time decision-making in large-scale settings. Conversely, heuristic approaches and periodic reallocation strategies often sacrifice solution quality for speed, leading to suboptimal resource utilization or butterfly effects, where local perturbations can cascade into system-wide reorganization (Li et al., 2024; Zhao et al., 2022).

Although recent advancements in deep reinforcement learning offer promising end-to-end responsiveness, they frequently struggle with interpretability and generalization when faced with abrupt changes in the dimensionality of the state space (Li et al., 2025). Thus, there exists a pressing need for a framework that can marry the speed of learning-based inference with the precision of combinatorial optimization.

In this paper, we propose a generative real-time mission planning framework designed to resolve multi-robot contingencies within seconds, offering responsiveness and scalability. The proposed framework enables rapid event-triggered replanning, achieving sub-second responses in small-scale scenarios and seconds-level responses in large-scale settings. Unlike conventional methods that restart optimization from scratch, our approach treats contingency handling as a prior-driven generative search process, where deep spatiotemporal priors are used to generate feasibility boundaries, cost intervals, and a constrained decision subspace that empirically reduces the effective search space for subsequent optimization. We introduce a hierarchical architecture that decouples the problem into dimension reduction and precise execution. At the macro level, a subtraction-average-based optimizer (SABO) is employed to autonomously tune a long short-term memory (LSTM) network. The deep prior mechanism rapidly extracts latent features from the system’s state flow, mapping discrete contingency events to continuous cost intervals and effectively compressing the search space by filtering out infeasible regions. Within this refined subspace, we deploy a guided whale optimization algorithm (GWOA) for plan regeneration. By integrating target-gap adaptive control with dimension-wise opposition learning, the GWOA overcomes the premature convergence typical of standard meta-heuristics, balancing global exploration with local exploitation. We rigorously validate this framework across six distinct contingency scenarios. Simulation results demonstrate that our model achieves robust reconfiguration within seconds and maintains operational stability even in large-scale stress tests. This work establishes an effective paradigm for resilient multi-robot systems coordination, demonstrating that the synergy of deep learning priors and evolutionary optimization can alleviate the practical dimensionality burden by empirically reducing the effective search space in stochastic environments.

## 2 Related work

Research on the orchestration of multi-robot missions in stochastic environments has evolved from static optimization toward adaptive and learning-based approaches for handling dynamic uncertainties (Choudhury et al., 2021). Early methodologies for multi-robot task allocation often framed mission planning as a static optimization problem, frequently utilizing variants of the vehicle routing problem or the multiple traveling salesman problem (Antonyshyn et al., 2023). However, as the number of robots and tasks increased, the computational complexity escalated, making these exact solutions impractical due to the curse of dimensionality (Chakraa et al., 2023). To address dynamic environments, researchers have introduced periodic reallocation and rolling-horizon frameworks (Ghassemi and Chowdhury, 2022). While these methods offer some adaptability, they often exhibit planning inertia, where the time required for re-computation of a new global optimum can exceed the operational window of a contingency, especially in high-entropy scenarios like robot failures or risk-induced mission terminations (Faruq et al., 2024).

Classical MRTA methods (Athira and Divyan, 2024), including rolling min-cost flow (R-MCF), assignment-based optimization (R-Assign) (Yu et al., 2025), and OR-Tools routing (ORT), typically formulate reallocation as an exact or combinatorial optimization problem. While yielding high-quality solutions under static assumptions, their reliance on repeated global optimization incurs prohibitive computational overhead during rapid contingencies. Alternatively, decentralized market-based methods (e.g., auction MRTA (Martin et al., 2026) improve scalability but often compromise global coordination when handling tightly coupled resources or sudden task injections. Contingency-aware and rolling-horizon planners enhance adaptability by reactively updating plans; however, they still reconstruct and solve computationally heavy problems, introducing planning inertia during high-frequency disruptions. Learning-based and hybrid MRTA methods (El-Afifi et al., 2025) seek to reduce replanning latency by using learned policies, neural predictors, or heuristic warm-starts. Nevertheless, most existing approaches mainly use learning modules for initialization, cost estimation, or direct policy approximation, without explicitly compressing the combinatorial search space or embedding learned feasibility structures into the optimization process.

To address these limitations, the proposed framework introduces a prior-guided generative subspace compression mechanism. Rather than solving the full MRTA problem or relying on simple warm-starts, the predictive module establishes feasibility-aware cost intervals to construct a reduced decision subspace. Guided evolutionary optimization is then executed within this constrained space, inherently preserving global coordination while ensuring real-time responsiveness under highly dynamic contingencies.

Meta-heuristic and evolutionary optimization techniques have emerged to overcome the limitations of exact solvers, offering gradient-free and robust exploration capabilities (Nassef et al., 2023; Houssein et al., 2024). Algorithms such as particle swarm optimization (PSO) and the whale optimization algorithm (WOA) have been widely adopted for multi-robot task allocation due to their effectiveness in exploring complex search spaces (Bansal and Aggarwal, 2024; Wang et al., 2024). The WOA, inspired by the bubble-net hunting behavior of humpback whales, has been shown to effectively locate global optima (Amador-Angulo and Castillo, 2023). However, standard meta-heuristics are prone to premature convergence and slow refinement, particularly in discrete, high-dimensional binary spaces (Becerra-Rozas et al., 2022). Recent advancements have sought to enhance the diversity and exploration capabilities of these algorithms through methods like chaotic maps and Lévy flights (Amador-Angulo and Castillo, 2023). Despite these improvements, meta-heuristics often perform undirected searches, failing to leverage historical system-state features. This can lead to redundant computations when the mission topology changes stochastically. Hybrid approaches, such as the combination of PSO and WOA, have been explored to mitigate these limitations in various optimization problems, including task workflow scheduling in cloud-fog computing environments (Bansal and Aggarwal, 2024). The integration of Interval Type-2 Fuzzy Logic Systems with WOA has also been proposed to dynamically adjust parameters, aiming for more robust performance (Amador-Angulo and Castillo, 2023).

The increasing prominence of deep reinforcement learning has introduced the paradigm of end-to-end mission planning. DRL offers significant responsiveness, enabling robots to adapt to dynamic environments and make real-time decisions (Hersi and Divya Udayan, 2024). For example, a deep reinforcement learning architecture based on a mamba attention model has been proposed to solve the problems with deadlines and workload constraints by capturing global dependencies among task points (Zhong et al., 2024). Furthermore, improved proximal policy optimization algorithms have been developed to enhance inter-robot collaboration and adaptability in dynamic settings (Li et al., 2025).

While deep reinforcement learning offers remarkable adaptiveness, its black-box nature and susceptibility to distribution shifts can limit its reliability in safety-critical missions (Mahmud et al., 2026). An emerging area involves physics-informed or prior-guided learning, where neural networks predict feasibility boundaries or cost intervals to warm-start traditional optimizers (Hoyos et al., 2025). Long short-term memory networks have shown efficacy in extracting spatiotemporal dependencies from robot trajectories, which is crucial for understanding dynamic behaviors and predicting future states (Ambuj et al., 2024). However, tuning such complex models often remains a bottleneck, requiring significant manual intervention and expert knowledge (Hersi and Divya Udayan, 2024). The development of frameworks for centralized management of contingency tasks and the generation of recommendations for task reallocation are critical for enhancing multi-robot system resilience (Dhanaraj et al., 2025). Contingency-aware task allocation and scheduling approaches, often employing hindsight optimization, proactively account for potential task failures and uncertainties, improving teaming efficiency by avoiding reactive addressing of issues (Dhanaraj et al., 2024). In human-supervised multi-robot missions, automated generation of task reallocation suggestions assists human operators in rapid decision-making, particularly when facing intermittent communications, delayed information, or robot failures. This is vital for complex tasks in dynamic and uncertain environments such as disaster response, where tasks may have deadlines and robots have range and payload constraints. Other research focuses on learning-based approaches to handle priority constraints and uncertainty in task allocation, seeking to overcome the limitations of traditional heuristic algorithms in dynamic programming environments (Deng et al., 2022).

Despite the progress in both deep learning-based planning and meta-heuristic optimization, directly applying a single paradigm to dynamic multi-robot allocation remains challenging. From a theoretical perspective, the contingency-driven task allocation problem is a high-dimensional NP-hard combinatorial optimization problem whose search space grows exponentially with the number of agents and tasks $|\Omega |=2^{MN}$ , where M denotes the number of agents and N denotes the number of tasks. Pure evolutionary algorithms must therefore explore an exponentially large decision space, leading to slow convergence. On the other hand, end-to-end learning approaches (e.g., reinforcement learning or transformer-based architectures) must approximate the entire mapping $\pi :S_{t}\to X$ , where $S_{t}$ is the system state and X is the assignment matrix. Learning such a mapping becomes unstable when the dimensionality of the state space changes due to contingency events. These limitations motivate the use of a two-stage architecture, where deep learning estimates feasible solution boundaries and evolutionary optimization performs fine-grained search within the reduced decision space.

Building on these insights, our work focuses on developing a framework for the centralized management of contingency tasks that generates real-time reallocation recommendations. The main contribution is not the individual components, but the hierarchical integration of deep prior estimation and evolutionary optimization into a unified decision pipeline for dynamic search-space reconstruction. Existing hybrid methods typically employ neural predictors to warm-start meta-heuristic optimizers by providing an initial solution or heuristic estimate. In contrast, the proposed framework generates feasibility-aware decision manifolds and cost priors. This enables the optimizer to operate within a dynamically constrained subspace rather than the full combinatorial domain. In this paper, the term generative is used in a general algorithmic sense to describe the generation of prior-guided search structures in the planning process. It does not refer to generative AI models such as GANs, VAEs, or large language models.

## 3 Problem formulation

### 3.1 Modeling of multi-robot missions and constraints

The mission is driven by a dynamic stream of time-sensitive orders. Unlike traditional static allocation, we define the order processing mechanism as an interactive agent-claiming and center-dispatching protocol. In this workflow, upon the arrival of a task, the central decision module broadcasts the requirements to all agents. Subsequently, agents autonomously evaluate their local states to screen feasible tasks and submit execution applications to the center. The decision module then aggregates these valid applications to execute global planning. This closed-loop feedback mechanism significantly enhances system robustness by filtering out infeasible assignments at the agent level before global optimization. The overall system scenario and multi-agent task definition in the dynamic mission environment are illustrated in Figure 1.

**FIGURE 1:**
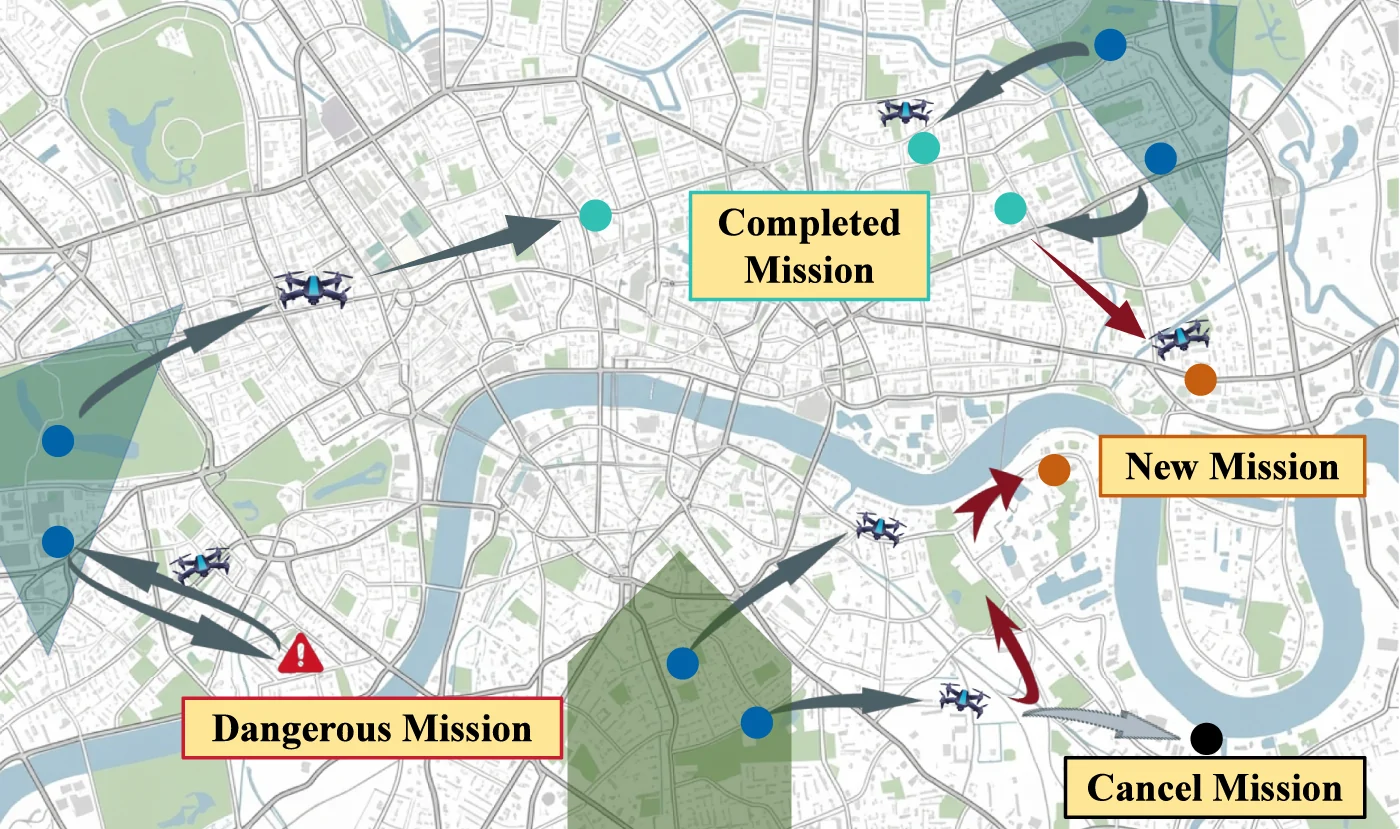
System scenario and multi-agent task definition in the dynamic mission environment.

Consider a heterogeneous multi-robot system comprising a set of agents denoted by $U=\left\{u_{1},u_{2},\ldots ,u_{M}\right\}$ . Each agent $u_{i}$ is characterized by a tuple $A_{i}=\left(p_{i},c_{i},v_{i}\right)$ , representing its current position, capability vector, and maximum velocity, respectively. Among these, the capacity vector represents the quantity of different resources transported in different volumes. Formally, let $O\left(t\right)=\left\{o_{1},o_{2},\ldots ,o_{N}\right\}$ denote the set of active task orders accepted by the decision module at time t . Each order $o_{j}$ is defined as a tuple $o_{j}=\left(l_{j},q_{j},\left[t_{j}^{s},t_{j}^{e}\right],w_{j},\lambda_{j}\right)$ , where $l_{j}$ is the target location, $q_{j}$ denotes the specific resource or capability requirement, $\left[t_{j}^{s},t_{j}^{e}\right]$ defines the strict time window for execution, enforcing the time-sensitivity, $w_{j}$ represents the priority weight of the task, $\lambda_{j}\in \left[0,1\right]$ indicates the intrinsic risk level associated with the task.

Let $X\left(t\right)\in {\left\{0,1\right\}}^{M\times N}$ be the decision matrix at time t , where $x_{ij}=1$ indicates the assignment of agent $u_{i}$ to order $o_{j}$ , provided that $u_{i}$ has submitted a valid claim for $o_{j}$ .

The fundamental constraints are modeled to ensure both physical feasibility and logical consistency. The capability constraint ensures that an agent assigned to a task possesses sufficient resources. Formally, $x_{ij}q_{j,k}\leq c_{i,k},\forall i,j,k$ , where $c_{i,k}$ is the agent’s available capacity for resource type k specified in its capability vector $c_{i}$ , $q_{j}={\left[q_{j,1},q_{j,2},\ldots ,q_{j,K}\right]}^{⊤}\in R_{+}^{K}$ and $c_{i}={\left[c_{i,1},c_{i,2},\ldots ,c_{i,K}\right]}^{⊤}\in R_{+}^{K}$ . In addition to physical capacity, the exclusivity constraint prevents resource conflict by ensuring that tasks are atomic and agents are not double-booked; this is modeled by $\sum_{i=1}^{M}x_{ij}\leq 1$ and $\sum_{j=1}^{N}x_{ij}\leq 1$ , implying a one-to-one mapping between available agents and active tasks. Furthermore, the system must adhere to the time window constraint, where the arrival time $\tau_{ij}$ constitutes a hard constraint determined by current time $t_{n}$ and travel velocity $v_{i}$ , satisfying $t_{j}^{s}\leq \tau_{ij}=t_{n}+\frac{\left|p_{i}-l_{j}\right|}{v_{i}}\leq t_{j}^{e}$ .

### 3.2 Taxonomy of dynamic contingencies model

Unlike static vehicle routing problems, the system state $S_{t}=\left(U_{t},O_{t}\right)$ is subject to stochastic transitions triggered by contingency events. We define a contingency event set $E=\left\{e_{r},e_{i},e_{l},e_{n},e_{c},e_{f}\right\}$ . The occurrence of any event e∈E triggers a generative reallocation process $S_{t}\to S_{t+\Delta }$ . In this work, the term generative does not refer to generating a complete task allocation directly. Instead, the deep prior module generates a structured optimization subspace and prior-guided seed population, which significantly reduces the dimensionality of the combinatorial search space before meta-heuristic optimization. We mathematically formalize the six contingency scenarios as follows.

- Risk-induced termination $\left(e_{r}\right)$ . Orders identified as high-risk during execution must be immediately terminated to preserve agent safety. The task set is reduced by a risk threshold filter $\theta_{r}$ ,

$$O_{t+\Delta }=O_{t}\backslash \left\{o_{j}|\lambda_{j}\left(t\right)>\theta_{r}\right\},$$

where \ is the set operation symbol for difference, indicating the removal of elements exceeding the risk threshold.

- Relevance loss $\left(e_{l}\right)$ . Due to dynamic environmental updates, certain tasks may lose their operational value. Let $V\left(o_{j},t\right)$ be the real-time value function,

$$O_{t+\Delta }=O_{t}\backslash \left\{o_{j}|V\left(o_{j},t\right)\approx 0\right\}.$$

- Constraint mutation $\left(e_{c}\right)$ . The operational constraints of existing orders may change abruptly, such as flight time window shortened. This is modeled as a parameter update map Φ ,

$$\exists o_{j}\in O_{t},\text{ s.t. }\left[t_{j}^{s},t_{j}^{e}\right]\overset{\Phi }{\to }\left[t_{j}^{\prime s},t_{j}^{\prime e}\right],$$

where $\left|t_{j}^{\prime e}-t_{j}^{\prime s}|\ll |t_{j}^{e}-t_{j}^{s}\right|$ .

- Dependency injection $\left(e_{n}\right)$ . New high-priority orders or dependency tasks $o_{w}$ are injected into the pool, expanding the task set dimension,

$$O_{t+\Delta }=O_{t}\cup \left\{o_{w}^{1},\ldots ,o_{w}^{k}\right\},$$

where requires a re-dimensioning of the decision matrix X from M×N to M×(N+k) .

- Criticality escalation $\left(e_{i}\right)$ . Certain standard tasks are re-designated as critical missions, requiring prioritized resource allocation. This implies a discrete jump in the weight parameter,

$$w_{j}\left(t+\Delta \right)=\alpha \cdot w_{j}\left(t\right),\;\alpha \gg 1.$$

- Agent paralysis $\left(e_{f}\right)$ . One or more agents suffer from hardware failure or neutralization. The available resource set contracts,

$$U_{t+\Delta }=U_{t}\backslash \left\{u_{f}\right\}.$$

Any task $o_{j}$ previously assigned to $u_{f}$ (i.e., $x_{f,j}=1$ ) must be released back to the unassigned pool $O_{p}$ .

### 3.3 The generative reallocation objective function

When a contingency e∈E occurs, the goal is not merely to minimize cost, but to generate a new feasible schedule $X^{\prime }$ that balances mission success, cost efficiency, and solution stability. We formulate this as a constrained optimization problem with penalty terms, as shown in Equation 1.

$$\underset{X^{\prime }}{\min}\;J=J_{e}+\omega J_{s}.$$ (1)

Here, $J_{e}$ denotes the execution cost, which captures both efficiency and reliability-related operational cost during task execution, including energy consumption, time expenditure, and risk-related penalties (intrinsic system or aerial vehicle factors). Moreover, $J_{s}$ denotes the stability penalty term, which measures the deviation between the newly generated allocation and the previous plan, thereby preventing excessive replanning-induced disruption. Additionally, ω serves as a scaling factor to regulate the magnitude of the penalty term $J_{s}$ , thereby controlling its contribution to the overall objective function. Unlike traditional vehicle routing problem formulations that strictly minimize travel time, our problem requires balancing energy conservation with delivery success probability within a rigid time window $\left[t_{j}^{s},t_{j}^{e}\right]$ . Mere speed is insufficient, while the resources need to be delivered reliably and efficiently. Therefore, we redefine the execution cost as a composite metric integrating energy expenditure and delivery failure penalties, as given in Equation 2.

$$J_{e}=\underset{i\in U_{t+\Delta }}{\sum }\underset{j\in O_{t+\Delta }}{\sum }n_{ij}x_{ij}^{\prime }\cdot \left(\beta_{1}\cdot E_{ij}\left(v_{o}\right)+\beta_{2}\cdot \Phi_{\Delta }\left(\tau_{ij}\right)+\beta_{3}\cdot \left(1-P_{s}^{ij}\right)\right),$$ (2)

where $E_{ij}\left(v_{o}\right)$ represents the minimum energy consumption required for agent $u_{i}$ to complete task $o_{j}$ under an optimal velocity profile $v_{o}$ , ensuring arrival within the specified time window while avoiding excessive high-speed energy consumption. Then $n_{ij}$ denotes the number of agents $u_{i}$ required to complete task $o_{j}$ . Meanwhile, $\Phi_{\Delta }\left(\tau_{ij}\right)$ is a soft penalty function for time window deviation. While hard constraints enforce feasibility, this term drives the solution toward the center of the time window to buffer against stochastic delays. Moreover, $P_{s}^{ij}$ denotes the probability of failure caused by intrinsic system or aerial vehicle factors, such as hardware malfunction, actuator failure, or onboard system degradation, which affects the feasibility of executing task j by agent i . Additionally, $\beta_{1},\beta_{2},\beta_{3}$ are weighting coefficients balancing energy, punctuality, and reliability. To ensure consistency across all comparative experiments, we set $\beta_{1}=\beta_{2}=\beta_{3}=1$ in the following simulations.

Then $n_{ij}$ denotes the number of agents $u_{i}$ required to complete task $o_{j}$ , which is defined in Equation 3.

$$n_{ij}=-\underset{j\in O_{t+\Delta }}{\sum }\log\left(1-\underset{i\in U_{t+\Delta }}{\prod }\left(1-{\left(1-p_{ij}\right)}^{x_{ij}^{\prime }}\right)\right).$$ (3)

Here, $\left(1-p_{ij}\right)$ represents the success rate of a single agent in response to environmental influences. This term ensures high reliability for critical tasks under contingencies.

Finally, the stability penalty term *J*_*s*_ is defined in Equation 4. To avoid butterfly effects, we penalize deviations from the original plan $X_{p}$ ,

$$J_{s}=\max\left(0,-\underset{i}{\sum }\underset{j}{\sum }\left(x_{ij}^{\prime }-x_{ij}^{p}\right)\cdot I\left(o_{j}\notin O_{A}\right)\right),$$ (4)

where I(⋅) is an indicator function that protects non-affected tasks from unnecessary reassignment, and $O_{A}$ denotes the affected set.

This optimization problem is NP-hard and time-sensitive. Standard solvers cannot meet the real-time requirement when the dimension of X changes dynamically. This necessitates the deep prior-guided generative framework proposed in Section 3.

## 4 Hierarchical generative planning framework

### 4.1 Framework overview

To address the NP-hard complexity and real-time requirements of the contingency-driven scheduling problem formulated in Section 2, we propose a hierarchical generative planning framework. Unlike periodic reallocation approaches, this framework is driven by an event-triggered, asynchronous mechanism. Specifically, the framework decomposes the global optimization problem into three coupled layers, each serving a distinct function in the generative process. The novelty lies in this three-stage coupling mechanism rather than individual modules.

The training-set generation framework produces diverse contingency scenarios by randomly perturbing agent states, task attributes, and environmental conditions. These simulated scenarios form the offline training dataset for the LSTM-based deep prior predictor. Then the macro-generation layer focuses on deep prior prediction, utilizing the subtraction average based optimizer (SABO) LSTM network to rapidly estimate the feasibility boundaries and cost intervals of the residual system resources. Next, the meso-pruning layer implements search space compression, which filters out invalid decision dimensions and reduces computational complexity based on the generated priors. It also synthesizes a prior-guided seed population, which is transmitted to the subsequent optimization module to initialize the whale optimization algorithm, thereby ensuring a high-quality starting point for the local search. Finally, the micro-execution layer handles plan regeneration, employing the guided whale optimization algorithm (GWOA) to precisely solve for the optimal re-allocation matrix within the refined subspace. The architectural interaction of these components is illustrated in Figure 2.

**FIGURE 2:**
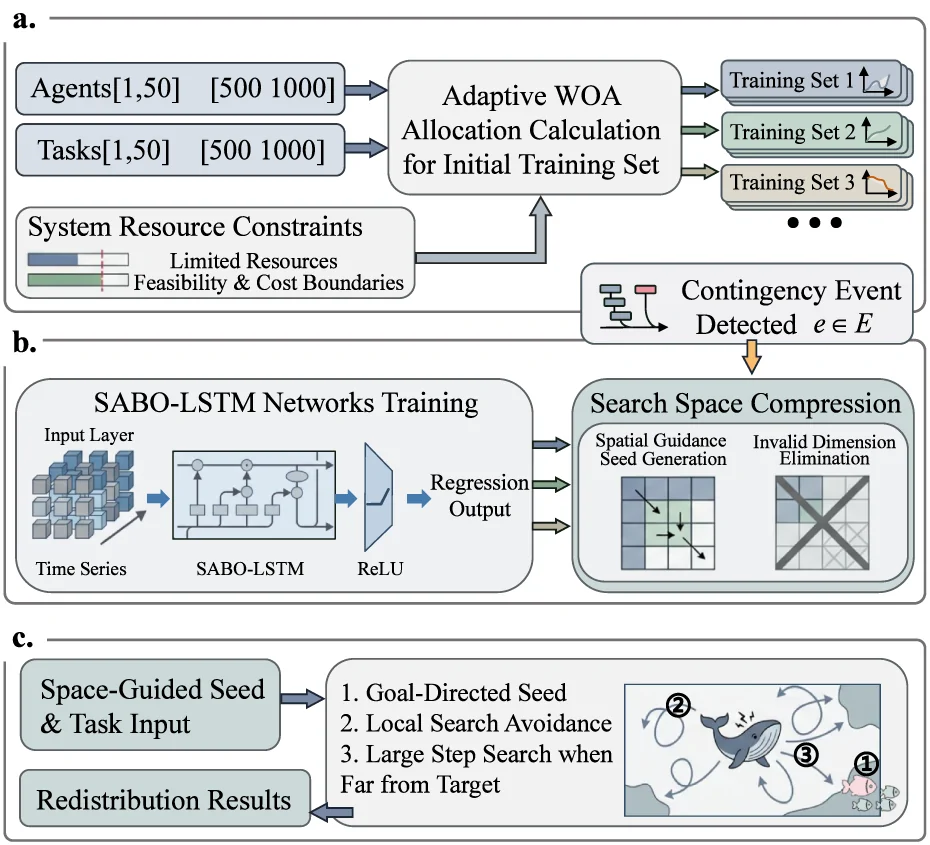
Overall framework of the proposed GWOA-enhanced SABO-LSTM predictive optimization system, integrating data processing, learning, and optimization modules. **(a)** Training set generation framework, **(b)** Deep prior prediction network, **(c)** Guided whale optimization algorithm.

The core philosophy of the proposed framework is to avoid exhaustive global searches when the system state topology changes. Let Ψ denote the generative reallocation operator. The process is strictly activated upon the detection of a contingency event e∈E .

The deep prior module uses an LSTM network with hidden dimension H and sequence length T . Before LSTM inference, the raw system state is normalized, reshaped, and padded into the predefined input representation $X_{\text{in}}\in R^{T\times f}$ . The computational cost of this state-encoding process may depend on the numbers of agents and tasks, particularly for features describing agent–task relationships. Given the encoded input with fixed T and f , the complexity of a single LSTM forward pass can be approximated as $O\left(TH^{2}\right)$ . The search space compression stage operates on the assignment matrix $X\in {\left\{0,1\right\}}^{M\times N}$ , where M denotes the number of agents and N the number of tasks. The pruning process evaluates feasibility conditions for each agent–task pair, resulting in a complexity of O(MN) which scales with the problem dimension.

The optimization stage employs the Guided Whale Optimization Algorithm (GWOA). Let P denote the population size and $T_{\max}$ the maximum iteration number. The complexity of the optimization process can be approximated as $O\left(P\cdot T_{\max}\cdot MN\right).$ Since the proposed framework performs prior-guided pruning before optimization, the effective search space dimension is significantly reduced to $M\times N^{\prime }$ , where $N^{\prime }<N$ . Consequently, the practical optimization cost becomes $O\left(P\cdot T_{\max}\cdot MN^{\prime }\right),$ which is substantially smaller than solving the original problem directly. The proposed framework reduces the effective search dimension through prior-guided pruning before optimization. However, this reduction should be interpreted as an empirical search-space compression mechanism rather than a theoretical elimination of the exponential worst-case complexity of the original combinatorial allocation problem.

The reduction from N to $N^{\prime }$ is determined by the specific contingency type, redistribution situation, and environmental constraints. Therefore, $N^{\prime }$ is not constant across different problem scales. In favorable cases, prior-guided pruning removes a large number of infeasible or low-quality task dimensions, leading to $N^{\prime }\ll N$ . However, in dense or highly constrained scenarios, the pruning ratio may decrease, and the effective search space may remain close to the original one.

In the worst case, if a contingency event triggers minimal pruning, the retained task dimension satisfies $N^{\prime }\approx N$ . Under this condition, the optimization-stage complexity degenerates from $O\left(P\cdot T_{\max}\cdot MN^{\prime }\right)$ to $O\left(P\cdot T_{\max}\cdot MN\right)$ . Accordingly, this worst-case bound clarifies that the proposed framework provides an empirical search-space reduction mechanism rather than a theoretical guarantee of near-linear scalability in all contingency scenarios.

In addition, compared with LSTM-GWOA, exact solvers such as mixed-integer programming approaches exhibit exponential worst-case complexity $O\left(2^{MN}\right)$ , making them impractical for real-time large-scale multi-robot systems. In deep reinforcement learning approaches, the action space grows combinatorially with the number of agents and tasks, which significantly increases training complexity and limits scalability. The proposed framework does not assume strict linear computational complexity. The complexity analysis reflects modular computational costs under fixed settings, while key parameters such as $N^{\prime }$ , P , and $T_{\max}$ are adaptive to problem scale. Accordingly, the observed behavior should be interpreted as a scalable complexity reduction mechanism rather than a linear asymptotic guarantee.

Formally, the framework maps the post-event state $S_{t+\Delta }$ to a new optimal decision matrix $X^{\prime }$ via a cascade of mappings, mathematically expressed as $X^{\prime }=\Psi \left(S_{t+\Delta }\right)=f_{1}\left(f_{2}\left(f_{3}\left(\phi \left(S_{t+\Delta }\right)\right)\right)\right)$ . This operation relies on an asynchronous event-triggered mechanism where a state monitor daemon continuously supervises system logic. Upon the detection of any contingency $e\in \left\{e_{r},\ldots ,e_{f}\right\}$ , the execution of the current plan Xp is immediately suspended, and the state feature vector $\phi \left(S_{t+\Delta }\right)$ is extracted to trigger the hierarchical reallocation process.

### 4.2 Deep prior prediction via SABO-LSTM

In the event of high-entropy contingencies, the system must rapidly estimate the feasibility boundaries of the residual resources. We employ a LSTM network to map the current discrete state sequence to continuous decision priors, where the deep prior predictor is implemented using an LSTM regression model rather than a probabilistic generative model. However, LSTM performance is highly sensitive to its hyperparameters. To address this, we propose the SABO-LSTM model, where the SABO is utilized to autonomously optimize the network architecture and training configuration. The LSTM-based predictor is trained offline using the generated contingency dataset and is used only for online inference during real-time task allocation. The detailed SABO-optimized LSTM regression framework for deep prior prediction is illustrated in Figure 3.

**FIGURE 3:**
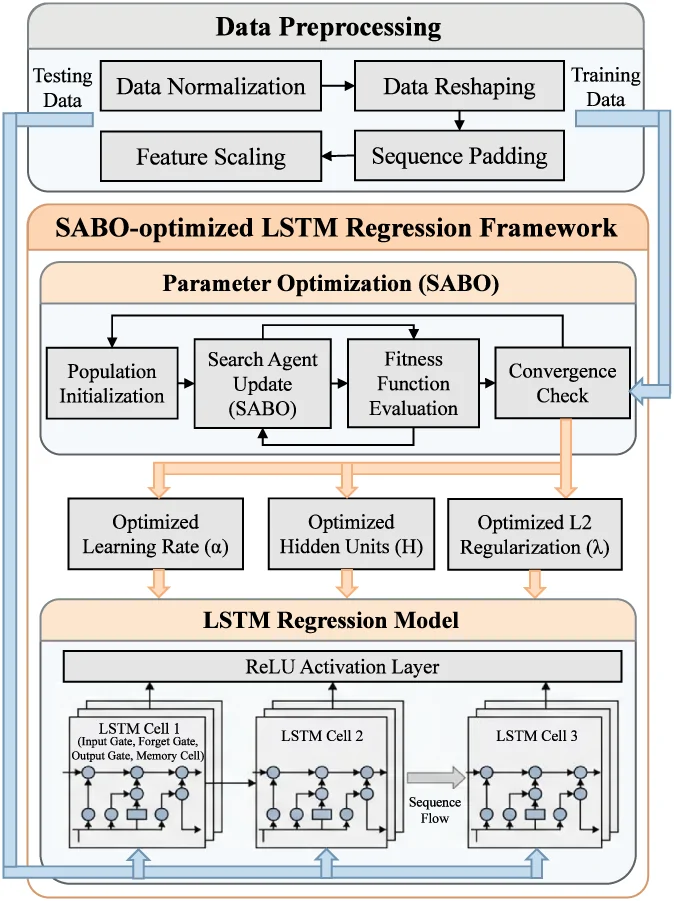
SABO-optimized LSTM regression framework for deep prior prediction, including data preprocessing, SABO-based hyperparameter optimization, and LSTM regression architecture design.

The input feature sequence $Xin\in R^{T\times f}$ is extracted from the system state $S_{t+\Delta }$ . The network comprises a sequence input layer, an LSTM layer with $N_{\text{hidden}}$ units, a ReLU activation layer, and a fully connected regression layer. The forward propagation for the hidden state $h_{t}$ and cell state $c_{t}$ is governed by the standard gate mechanisms, forget gate $f_{t}$ , input gate $i_{t}$ , and output gate $o_{t}$ . The output vector $y_{\text{pred}}={\left[{\hat{J}}_{\text{min}},{\hat{J}}_{\text{max}},{\hat{R}}_{\text{min}}\right]}^{\text{T}}$ represents the predicted cost interval and reliability lower bound. To bridge prediction and optimization, a surrogate evaluation function is constructed from SABO-LSTM outputs. Specifically, given $\left[{\hat{J}}_{\min},{\hat{J}}_{\max},{\hat{R}}_{\min}\right]$ , the surrogate cost is defined as a risk-adjusted expectation in Equation 5.

$$\tilde{J}\left(X\right)=\frac{{\hat{J}}_{\min}+{\hat{J}}_{\max}}{2}.$$ (5)

Manual tuning of hyperparameters is computationally prohibitive for real-time systems. We formulate the hyperparameter tuning as a global optimization problem in Equation 6.

$$F\left(z\right)=\sqrt{\frac{1}{N_{v}}\overset{N_{v}}{\underset{i=1}{\sum }}{\left[y_{i}-f\left(x_{i},Train\left(T_{t},z\right)\right)\right]}^{2}},$$ (6)

where $z=\left[\eta ,N_{h},\lambda_{L2}\right]$ represents the decision vector containing the initial learning rate, hidden layer size, and L2 regularization coefficient, respectively. Moreover, $T_{t}$ is the training dataset and $Train\left(T_{t},z\right)$ represents the model obtained by training on $T_{t}$ with parameters z . Similarly, $T_{v}={\left\{\left(x_{i},y_{i}\right)\right\}}_{i=1}^{N_{v}}$ is the validation dataset comprising $N_{v}$ samples, in which $x_{i}$ is the feature vector of the i -th sample and $y_{i}$ is the corresponding ground-truth label.

We employ the SABO, a swarm-based meta-heuristic, to solve for z* . Unlike gradient-based methods, SABO updates the population positions using the arithmetic mean of subtraction differences between agents, which enhances robust exploration. Let $X_{i}\in R^{3}$ be the position of the i -th search agent, which represents a set of hyperparameters. The displacement vector $v_{i}$ is calculated by aggregating the difference between agent i and all other agents j , weighted by their fitness difference, as defined in Equation 7.

$$v_{i,d}=\overset{N_{\text{p}}}{\underset{j=1}{\sum }}\left(x_{j,d}-I\cdot x_{i,d}\right)\cdot sgn\left(F\left(x_{i}\right)-F\left(x_{j}\right)\right),$$ (7)

where I is a stochastic scaling factor $I\approx \left\lfloor 1+2r\right\rfloor$ . The corresponding position update rule is defined in Equation 8.

$$X_{i}^{n+1}=X_{i}^{n}+\frac{r⊙v_{i}}{N_{\text{p}}}.$$ (8)

Here, r is a random vector in ${\left[0,1\right]}^{3}$ , and ⊙ denotes the Hadamard product. This mechanism guides the hyperparameters towards the global optimum z* , ensuring the LSTM achieves minimal generalization error on the validation set $T_{v}$ .

Once the SABO-LSTM model is trained offline, its optimal parameters are frozen. During the online phase, the network takes the contingency state $\phi \left(S_{t+\Delta }\right)$ and instantly generates the prior vector P . To bridge the prediction module with the optimization stage, we further define a fast surrogate evaluator $J_{p}$ for any candidate allocation matrix X , which provides a low-cost estimate of its composite scheduling cost before full fitness evaluation. Specifically, *J*_*p*_ is computed as the midpoint of the predicted cost interval scaled by the predicted unreliability risk, as shown in Equation 9.

$$J_{p}=\frac{{\hat{J}}_{\min}+{\hat{J}}_{\max}}{2}\cdot \left(1-{\hat{R}}_{\min}\right).$$ (9)

It represents a risk-adjusted baseline cost used to guide the adaptive exploration–exploitation balance in GWOA, and serves as a reference for target-gap computation.

Figure 4 provides a detailed flowchart of the data interaction between the SABO-LSTM predictor and the GWOA optimizer, explicitly showing how the predicted priors are converted into search-space pruning and optimization guidance.

**FIGURE 4:**
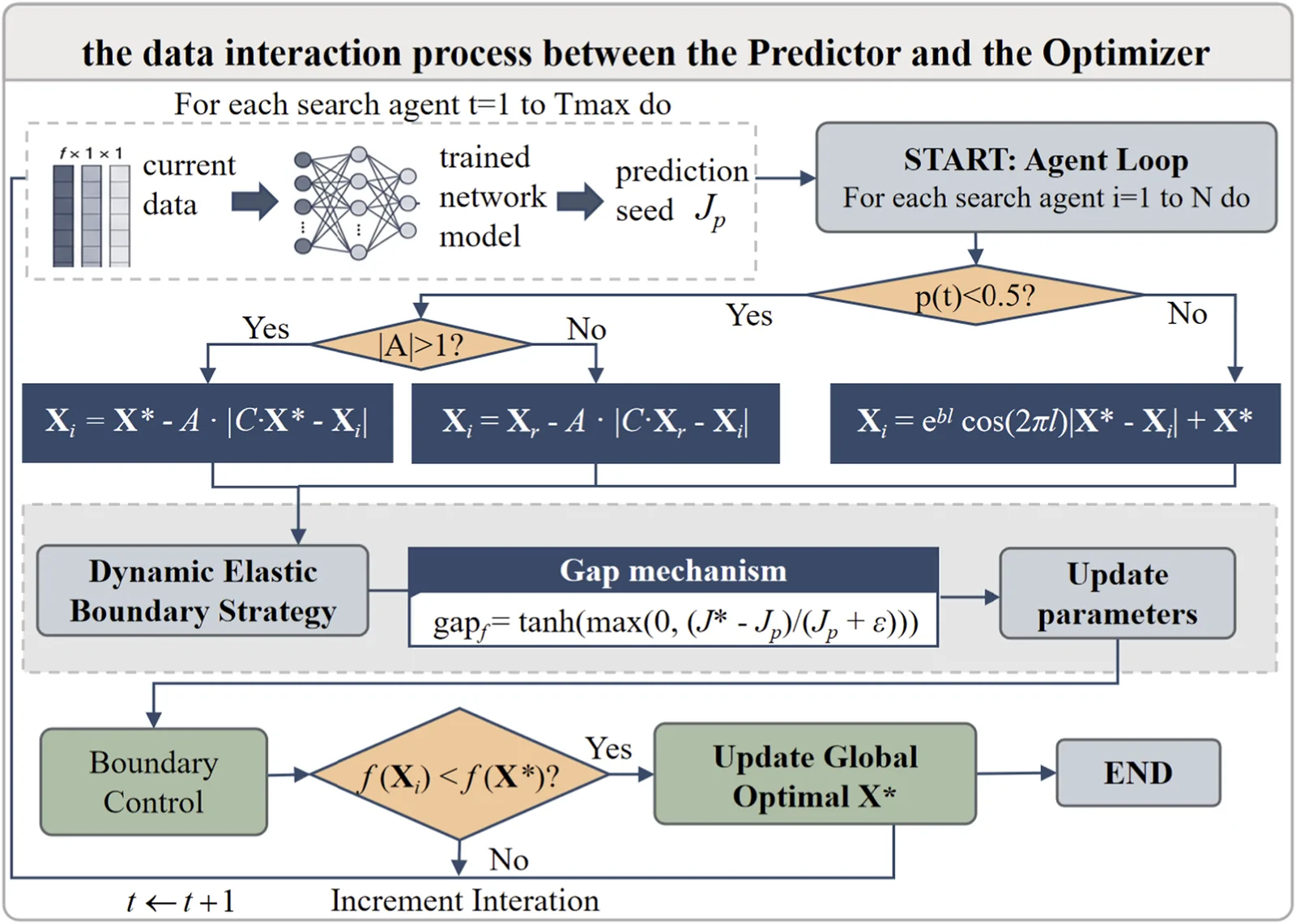
Interactive optimization mechanism between the SABO-LSTM predictor and GWOA optimizer for iterative performance enhancement.

The specific algorithm can be found in Algorithm 1, where $F_{i}^{\left(0\right)}$ , $F_{j}^{\left(0\right)}$ denote the fitness values at iteration t=0 . The displacement of agent i is defined as $\Delta X_{i}=X_{i}^{\left(n+1\right)}-X_{i}^{\left(n\right)}$ . The pairwise interaction term is explicitly defined as $\Delta V_{ij}\leftarrow \left(X_{j}-I\cdot X_{i}\right)\cdot \text{sgn }\left(F_{i}^{\left(0\right)}-F_{j}^{\left(0\right)}\right)$ , and the accumulation is initialized as $\Delta X_{i}=0$ .

Algorithm 1 SABO-LSTM Hyperparameter Optimization and Prior Generation.

 **Require:** Dataset D={P,T} , population size $N_{p}$ , max iterations $T_{\max}$ , bounds $\left[L_{b},U_{b}\right]$ , agent i , j , real-time contingency state vector $\phi \left(S_{t+\Delta }\right)$ .
 **Ensure:** Optimal network $H^{*}$ , decision priors P .
  1: initialize population $X\in R^{N_{p}\times 3}$ within $\left[L_{b},U_{b}\right]$

  2: $X_{i}=\left[\eta ,N_{h},\lambda_{L2}\right]$

  3: **for** i=1 to $N_{p}$ **do**
  4: $F_{i}^{\left(0\right)}\leftarrow \text{Train}\left(X_{i},D\right)$

  5: **end** **for**
  6: identify $X^{*}$ and $F^{*}$

  7: **for** t=1 to $T_{\max}$ **do**
  8: $\Delta X\leftarrow 0_{N_{p}\times 3}$

  9:    **for** i=1 to $N_{p}$ **do**
  10:    **for** j=1 to $N_{p}$ **do**
  11: I←round(1+2⋅rand)

  12: $\Delta V_{ij}\leftarrow \left(X_{j}-I\cdot X_{i}\right)\cdot \text{sign}\left(F_{i}^{\left(0\right)}-F_{j}^{\left(0\right)}\right)$

  13: $\Delta X_{i}\leftarrow \Delta X_{i}+\Delta V_{ij}$

  14:    **end** **for**
  15: $\tilde{X}\leftarrow X_{i}+\left(r\cdot \Delta X_{i}\right)/N_{p}$

  16:    Clip $\tilde{X}\in \left[L_{b},U_{b}\right]$

  17: $F_{i}^{\left(1\right)}\leftarrow \text{TrainEvaluateLSTM}\left(\tilde{X},D\right)$

  18:    **if** $F_{i}^{\left(1\right)}<F_{i}^{\left(0\right)}$ **then**
  19: $X_{i}\leftarrow \tilde{X}$ ; $F_{i}^{\left(0\right)}\leftarrow F_{i}^{\left(1\right)}$

  20:    **end** **if**
  21:    **if** $F_{i}^{\left(0\right)}<F^{*}$ **then**
  22: $X^{*}\leftarrow X_{i}$ ; $F^{*}\leftarrow F_{i}^{\left(0\right)}$

  23:    **end** **if**
  24:   **end** **for**
  25: **end** **for**
  26: $H^{*}\leftarrow X^{*}$

  27: $P\leftarrow \text{Predict}\left(H^{*},\phi \left(S_{t+\Delta }\right)\right)$

  28: **return** P

### 4.3 Contingency-aware search space compression

To achieve the responsiveness required for dynamic reallocation, the exponential search space $\Omega ={\left\{0,1\right\}}^{M\times N}$ needs to be effectively compressed before optimization. We construct a dynamic pruning filter that leverages the specific contingency type e and the deep priors P generated. This filtration geometrically contracts the original high-dimensional space into a significantly smaller and more tractable subspace $\Omega^{\prime }\subset \Omega$ . By intelligently discarding infeasible or low-quality solutions in advance, the computational burden on the subsequent iterative algorithm is drastically reduced. This pruning mechanism is designed as a structural reduction strategy guided by learned priors, rather than a handcrafted heuristic rule, and serves as a key component of the integrated generative framework.

In the proposed framework, pruning is not applied to complete solution vectors in an arbitrary manner, but is performed at two levels, i.e., agent-task assignment elements and candidate solution matrices during population evolution. Hard feasibility pruning first removes assignment variables $x_{ij}$ that violate deterministic constraints, including contingency-triggered task termination, parameter-shift-induced infeasibility, and capacity or time-window violations within GWOA evaluation, yielding the subset $\Omega_{r}^{\prime }$ . In parallel, soft probabilistic pruning leverages SABO-LSTM outputs $\left[{\hat{J}}_{\min},{\hat{J}}_{\max},{\hat{R}}_{\min}\right]$ , where a surrogate cost $J_{p}$ is used to identify candidates within the prediction-consistent region $\Omega_{p}$ ; low-probability solutions are down-weighted or probabilistically excluded rather than strictly removed. The final pruned space is defined as $\Omega^{\prime }=\Omega_{r}^{\prime }\cap \Omega_{p}^{\prime }\cap \Omega_{f}$ , where $\Omega_{r}^{\prime }$ , $\Omega_{p}^{\prime }$ , and $\Omega_{f}$ denote hard constraint removal, prediction-guided filtering, and physical feasibility constraints, respectively.

To avoid ambiguity, we explicitly emphasize that pruning is performed on agent–task assignment variables and population-level candidate solutions, rather than eliminating entire solution trajectories in a single deterministic step.

For contingencies necessitating immediate task termination, such as $e_{r}$ or $e_{l}$ , the filter applies a deterministic pruning strategy. In these scenarios, the decision variables associated with compromised or obsolete tasks are strictly locked to zero to ensure safety and operational efficiency. Mathematically, this exclusion is formalized in Equation 10.

$$\Omega_{r}^{\prime }=\left\{X\in \Omega |\lambda_{j}>\theta_{r},x_{ij}=0\right\}.$$ (10)

This constraint effectively removes specific dimensions from the optimization variables, preventing the algorithm from wasting computational resources on exploring options that have become physically or tactically invalid.

Conversely, for events involving parameter shifts like $e_{i}$ or $e_{c}$ , the framework employs a prior-guided probabilistic pruning mechanism. Utilizing the resource intensity vector $\hat{\rho }$ derived from the SABO-LSTM, the search scope is refined to retain reliable agent-task pairs. This logic dictates that agents identified as unreliable by the deep prior are pre-excluded from feasible assignments, defined by the set defined in Equation 11.

$$\Omega_{p}^{\prime }=\left\{X\in \Omega |x_{ij}=0\right\},$$ (11)

where ∀(i,j)∈S , S represents the set of filtered indices subject to the condition that if the predicted resource intensity ${\hat{\rho }}_{i}<\epsilon$ is classified as high, then the assignment variable $x_{ij}$ is strictly constrained to 0. By filtering out these low-probability allocations based on deep learning predictions, the search dimensionality is significantly reduced while preserving the most promising regions for the global optimum. Based on this, the final feasible search space is defined in Equation 12.

$$\Omega^{\prime }=\Omega_{r}^{\prime }\cap \Omega_{p}^{\prime }\cap \Omega_{f},$$ (12)

where $\Omega_{f}$ denotes feasibility constraints derived from environmental and system reliability factors. This formulation ensures that the GWOA operates strictly within a prediction-informed and feasibility-consistent subspace.

If the prediction error exceeds the δ -expanded boundary, the GWOA responds by broadening its exploration range, which may incur additional computational cost. This fallback mechanism reduces the risk of prematurely excluding potentially high-quality solutions and improves robustness against prediction errors.

To enhance robustness against prediction errors, the feasible region is enlarged using an adaptive tolerance δ , defined as $\delta =\kappa \left({\hat{J}}_{\max}-{\hat{J}}_{\min}\right)$ , where κ is a dimensionless tolerance coefficient selected offline using the validation set and subsequently fixed during online optimization. The coefficient is chosen to balance prediction robustness and search-space compression. Accordingly, a wider predicted interval results in a larger tolerance margin, whereas a narrower interval leads to a tighter prediction-guided search region. The resulting guidance region is defined in Equation 13.

$$\Omega_{p}=\left\{X|\tilde{J}\left(X\right)\in \left[{\hat{J}}_{\min}-\delta ,{\hat{J}}_{\max}+\delta \right]\right\}.$$ (13)

Here, $\Omega_{p}$ is the guidance region for the predicted direction, and δ is an adaptive uncertainty margin proportional to the prediction interval width. In such cases, the GWOA reverts to broader exploration within the original search space, thereby reducing the risk of excluding promising candidate solutions at the expense of increased computational cost.

The proposed pruning strategy is hybrid in nature, combining hard feasibility constraints and soft probabilistic filtering. Hard pruning is applied to deterministic constraint violations (e.g., contingencies necessitating immediate task termination and events involving parameter shifts), while soft pruning is governed by probability-based retention scores derived from environmental and system reliability priors, such as $\Omega_{p}$ . This design ensures both feasibility guarantee and exploration diversity.

Additionally, it should be clarified that, within the proposed framework, the deep prediction module provides a prior-guided reference cost level $J_{p}$ , which is used to construct the prediction-guided feasible region $\Omega_{p}$ . This component mainly guides the optimization direction by restricting the search space to high-probability regions.

In contrast, feasibility constraints are enforced at two levels. First, during the pruning stage, where infeasible or low-probability agent-task assignments are excluded from $\Omega_{p}^{\prime }$ . Second, within the GWOA optimization stage, where constraint violations are explicitly penalized in the fitness function.

### 4.4 Guided whale optimization for plan regeneration

The final stage is to pinpoint the optimal binary decision matrix $X^{*}$ within the pruned subspace $\Omega^{\prime }$ . Standard WOA operates in continuous space and lacks mechanisms to escape local optima in high-dimensional discrete constraints. We propose a guided whale optimization algorithm that integrates chaotic theory, Levy flight strategies, and a contingency-aware inheritance mechanism.

To clarify the algorithmic contribution of the proposed GWOA, it is designed as an enhanced whale optimization strategy tailored for discrete contingency-driven assignment problems, rather than a general-purpose metaheuristic. The proposed GWOA introduces several problem-driven enhancements over the standard whale optimization algorithm. First, Chebyshev chaotic mapping combined with prior information from the SABO-LSTM module is used to improve population initialization and enhance search diversity. Second, a target-gap–guided adaptive strategy is incorporated to dynamically balance exploration and exploitation according to the deviation between current and predicted optimal cost. Third, Lévy-flight perturbations with adaptive scaling are introduced to strengthen global exploration and prevent premature convergence. Fourth, a probabilistic stochastic rounding mechanism is adopted to preserve structural information when mapping continuous updates into discrete binary decisions. Finally, a dimension-wise opposition-based refinement strategy inspired by pinhole imaging is employed to improve local diversity and enhance escape capability from local optima. These mechanisms collectively improve the convergence efficiency and robustness of WOA in discrete combinatorial optimization problems.

To enhance the ergodicity of the initial population and avoid premature convergence, we replace standard pseudo-random generators with Chebyshev Chaotic Maps. Combining this with the P from the SABO-LSTM layer, the position of the i -th whale $x_{i}$ is initialized according to Equation 14.

$$x_{i,d}^{n}=\left.l_{b,d}+\left(u_{b,d}-l_{b,d}\right)\cdot \Psi_{\text{C}}\left(k,x_{i,d}^{n-1}\right)\right),$$ (14)

where $\Psi_{\text{C}}$ denotes the Chebyshev map defined by the recursive relation $x_{k+1}=\cos\left(k\cdot \arccos\left(x_{k}\right)\right)$ , mapping the search agents uniformly across the feasible cost intervals derived in the Macro layer.

Subsequently, an adaptive exploration–exploitation mechanism guided by an externally predicted target cost is introduced. Let J(t) denote the best fitness at iteration t and $J_{p}$ the predicted optimal cost. A normalized target gap is defined in Equation 15.

$$gap\left(t\right)=\max\left(0,\frac{J\left(t\right)-J_{p}}{J_{p}+\varepsilon }\right),$$ (15)

and mapped to [0, 1) using tanh(⋅) to avoid numerical instability. This gap dynamically regulates the balance between global exploration and local exploitation. Unlike standard WOA, the linear decay parameter a is modified according to Equation 16.

$$a\left(t\right)=\min\left(2,2-\frac{2t}{T_{\max}}+0.5\cdot \tanh\left(gap\left(t\right)\right)\right),$$ (16)

ensuring sustained exploration when the current solution is far from the predicted optimum. Meanwhile, the behavioral transition probability evolves according to Equation 17.

$$p\left(t\right)=0.3+0.5\cdot \frac{t}{T_{\max}},$$ (17)

favoring encircling and spiral exploitation in later stages. Then, we inject a Levy flight disturbance into the position update equation using a stability index β=0.7 to prevent stagnation in local optima, as formulated in Equation 18.

$$X^{n+1}\left(t\right)=X^{n}\left(t\right)+\alpha \cdot Levy\left(\beta \right)\cdot \left(X^{n}\left(t\right)-X^{*}\right),$$ (18)

where $Levy\left(\beta \right)∼\frac{u}{|v{|}^{1/\beta }}$ follows a heavy-tailed distribution and the perturbation weight α is adaptively scaled, $\alpha =0.1\cdot \left(1+5\cdot \tanh\left(gap\left(t\right)\right)\right)$ . Larger gaps induce stronger long-range jumps, while near-target states favor fine-grained local refinement.

Since robot task assignment is a discrete problem, $x_{i}\in \left\{0,1\right\}$ , but WOA operates in continuous space, simple rounding causes information loss. We employ a Probabilistic Stochastic Rounding method. For a continuous value $x_{c,i}$ , the discrete value $x_{d,i}$ is determined according to Equation 19.

$$x_{d,i}=\left\lfloor x_{c,i}\right\rfloor +I\left(r<\left(x_{c,i}-\left\lfloor x_{c,i}\right\rfloor \right)\right),$$ (19)

where I(⋅) is the indicator function. This preserves the gradient information of the continuous optimization process in a statistical manner.

Simultaneously, the limitations of capabilities and the constraints of time windows are calculated within the fitness function defined in Equation 20.

$$J\left(X\right)=J_{e}\left(X\right)+\omega J_{s}\left(X\right),$$ (20)

where ω=50 is a static penalty factor, based on experimental settings, enforcing the algorithm to converge strictly to feasible regions where resource demands do not exceed availability, $b_{l}\geq 0$ .

To further mitigate interdimensional coupling and enhance population diversity in high-dimensional search, we incorporate a dimension-wise opposition learning strategy inspired by the optical principle of pinhole imaging. Conventional opposition-based learning often treats the solution vector monolithically, which in high-dimensional spaces can lead to correlated perturbations that limit exploration. By analogizing each decision variable to a point source projected through a pinhole, the method decouples dimensions and generates refined, diversity-preserving candidate solutions.

Formally, let $x^{n+1}\left(t\right)$ denote the current best solution at iteration t , with upper and lower bounds $u_{b}$ and $l_{b}$ defined per dimension. For each dimension i , an opposition-based candidate ${\tilde{x}}_{i}$ is derived via the pinhole imaging transformation in Equation 21.

$${\tilde{x}}_{i}=\left(u_{b,i}+l_{b,i}\right)\left(\frac{1}{2}+\frac{1}{2\eta }\right)-\frac{x_{i}^{n+1},i}{\eta },$$ (21)

where η=1 corresponds to the image-to-object height ratio in a unit-scale pinhole model. This operation is applied independently across all dimensions, effectively performing a coordinated yet dimensionally decoupled refinement of the current best solution.

The generated candidate $\tilde{X}$ is then evaluated and retained only if it improves upon $x^{n+1}\left(t\right)$ . This mechanism not only refines the incumbent solution locally but also introduces structured diversity, increasing the probability of escaping local optima while preserving convergence precision. Integrated into the GWOA update cycle, the strategy acts as a lightweight, per-iteration enhancement that complements the global exploration provided by Lévy flights and the population diversity introduced by chaotic initialization.

The pseudocode of the GWOA can be found in Algorithm 2, where $J_{p}$ denotes the prior-estimated cost level generated by the deep learning module, which serves as a reference target for guiding the optimization process.

Algorithm 2 Guided Whale Optimization Algorithm.

 **Require:** Population size N , maximum iterations $T_{\max}$ , dimension D , bounds $\left[l_{b},u_{b}\right]$ , cost value C , cost parameters (C,N,S) , priors P , pruned space $\Omega^{\prime }$

 **Ensure:** Best binary solution $X^{*}$ and best fitness $J^{*}$

  1: $J^{*}\leftarrow +\infty$ , $X^{*}\leftarrow 0$ , $J_{p}\leftarrow \Omega^{\prime }\left(x_{i},P\right)$ ,
  2: **for** i=1 to N **do**
  3:  **for** d=1 to D **do**
  4: $x_{d}\leftarrow \cos\left(d\cdot \arccos\left(x_{d}\right)\right)$

  5: $x_{i,d}\leftarrow l_{b,d}+\left(u_{b,d}-l_{b,d}\right)\cdot x_{d+1}$

  6:  **end** **for**
  7: **end** **for**
  8: **for** t=1 to $T_{\max}$ **do**
  9: $J_{i}\leftarrow J\left(X_{i}|C,N,S\right)$

  10:  **if** $J_{i}<J^{*}$ **then**
  11: $J^{*}\leftarrow J_{i}$ , $X^{*}\leftarrow X_{i}$

  12:  **end** **if**
  13: ${\text{gap}}_{f}\leftarrow \tanh\;\left(\max\;\left(0,\frac{J^{*}-J_{p}}{J_{p}+\epsilon }\right)\right)$

  14:  **if** $gap_{f}<10^{-3}$ **then**
  15:    break
  16:  **end** **if**
  17: $a_{0}\leftarrow 2\left(1-\frac{t}{T_{\max}}\right)$

  18: $a\leftarrow \min\left(2,a_{0}+0.5\cdot {\text{gap}}_{f}\right)$

  19: $p\left(t\right)\leftarrow 0.3+0.5\cdot t/T_{\max}$

  20:  **for** i=1 to N **do**
  21: $A\leftarrow 2ar_{1}-a$ , $C\leftarrow 2r_{2}$

  22:   **if** p(t)<0.5 **and** |A|<1 **then**
  23: $X_{i}\leftarrow X^{*}-A\cdot |CX^{*}-X_{i}|$

  24:   **else** **if** p(t)<0.5 **and** |A|≥1 **then**
  25: $X_{i}\leftarrow X_{r}-A\cdot |CX_{r}-X_{i}|$

  26:   **else** **if** p(t)≥0.5 **then**
  27: $l∼U\left(a_{0}/2,1\right)$

  28: $X_{i}\leftarrow e^{bl}\cos\left(2\pi l\right)|X^{*}-X_{i}|+X^{*}$

  29:   **end** **if**
  30: $\alpha \leftarrow 0.1\cdot \left(1+5\cdot {\text{gap}}_{f}\right)$

  31: $X_{i}\leftarrow X_{i}+\alpha \cdot Levy\left(\beta \right)⊙\left(X_{i}-X^{*}\right)$

  32:   **for** d=1 to D **do**
  33: $X_{i}\leftarrow f_{c}\left(X_{i},l_{b},u_{b}\right)$

  34: $x_{i,d}\leftarrow \left\lfloor x_{i,d}\right\rfloor +I\left(rand<x_{i,d}-\left\lfloor x_{i,d}\right\rfloor \right)$

  35:   **end** **for**
  36:  **end** **for**
  37:  **if** t=20n **and** ${\text{gap}}_{f}>0.5$ **then**
  38: $J_{\max}\leftarrow l_{b}+\left(u_{b}-l_{b}\right)\cdot r_{3}$

  39:   **end** **if**
  40: **end** **for**
  41: **return** $X^{*},J^{*}$

## 5 Simulation experiments and analysis

In this section, we conduct extensive simulation experiments to validate the efficacy of the proposed hierarchical generative planning framework. The experiments are designed to evaluate three critical aspects, the prediction accuracy of the SABO-LSTM, the convergence efficiency of the GWOA optimizer, and the real-time responsiveness and stability of the system under contingency scenarios.

### 5.1 Experimental setup and dataset generation

The simulations are conducted on a PC with an Intel Core i9-14900K CPU and 32GB RAM, utilizing MATLAB R2024b. We generated synthetic datasets as outlined in Section 4, with the number of agents and tasks spanning three scales: small [0,20], medium [100,200], and large (up to 1000 tasks). At each scale, a meta-heuristic solver was used to generate 1,000 historical task allocation samples. Then, the samples were split into a 70% training set and a 30% test set for the SABO-LSTM model. For each problem scale, multiple scenario instances were generated under the corresponding agent-task configuration.

Each sample records the system-state feature vector ϕ(S) , which includes the number of active tasks, task priorities, spatial distances between tasks and robots, the number of available robots, robot successful-delivery probabilities, the baseline environmental complexity, and the intensity of the unexpected disturbance. For each system-state sample, the corresponding training target is defined as ${\left[J_{\min},J_{\max},R_{\min}\right]}^{T}$ , where $J_{\min}$ and $J_{\max}$ denote the minimum and maximum objective values obtained from the feasible allocation results generated under the same scenario, respectively, and $R_{\min}$ denotes the corresponding lower reliability bound. Thus, the offline training dataset consists of paired samples $\phi \left(S\right),{\left[J_{\min},J_{\max},R_{\min}\right]}^{T}$ , which are used to train the SABO-LSTM predictor to estimate cost and reliability priors for subsequent search-space compression.

In the considered application context, real-time responsiveness is defined relative to the contingency handling horizon of the mission system. For local or moderate-scale disruptions, sub-second replanning is desirable and achieved in our experiments. For large-scale emergency reallocation involving hundreds of agents and tasks, latency within several seconds to tens of seconds remains operationally acceptable, since the replanning cycle is still significantly shorter than the execution horizon of the affected missions.

To ensure a fair comparison, the population size is set to $N_{p}=30$ and the maximum iteration count $T_{\max}=200$ for all meta-heuristic algorithms. The specific parameters for the proposed framework, optimized via SABO, are listed in Table 1.

**TABLE 1:** SABO-LSTM hyperparameter optimization range.

Hyperparameter	$l_{b}$	$u_{b}$
Learning rate (η)	$1\times 10^{-4}$	$1\times 10^{-2}$
Number of hidden layer nodes $\left(N_{h}\right)$	10	100
L2 regularisation $\left(\lambda_{L2}\right)$	$1\times 10^{-6}$	$1\times 10^{-4}$

### 5.2 Experimental prediction model validation and comparative analysis

To comprehensively evaluate the efficacy and generalization capability of our model in capturing complex non-linear patterns, and to elucidate the driving mechanisms underlying its predictions, we systematically validated the performance across three dimensions: goodness-of-fit, error distribution, and feature interpretability. Figure 5 illustrates the comparative regression performance between training and testing sets, underscoring the fitting robustness across distinct data distributions. Subsequently, Figure 6 quantifies the central tendency of predictive deviations and the model’s tracking accuracy for dynamic variations through an analysis of error histograms superimposed with local prediction trajectories. Finally, to clarify the influence weights of critical variables, Figure 7 presents SHAP-based feature dependency plots, revealing the intrinsic correlations between the top three dominant features and the prediction target.

**FIGURE 5:**
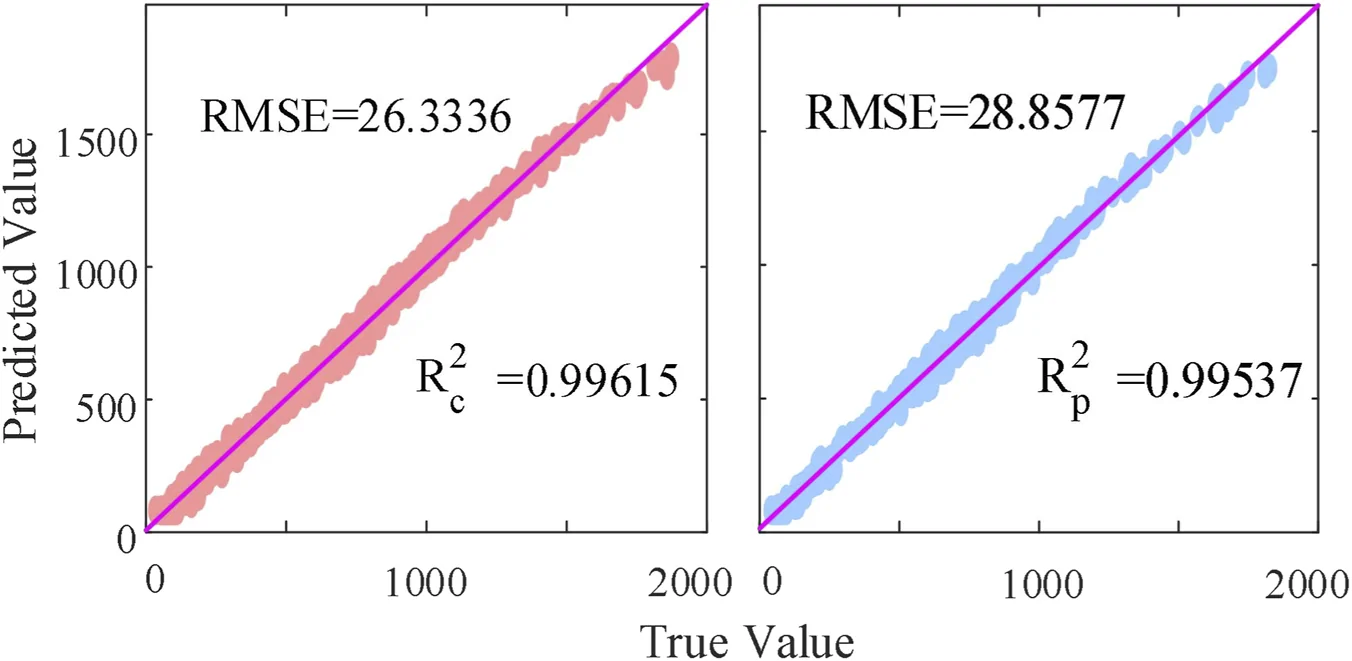
Performance comparison chart between training and test sets.

**FIGURE 6:**
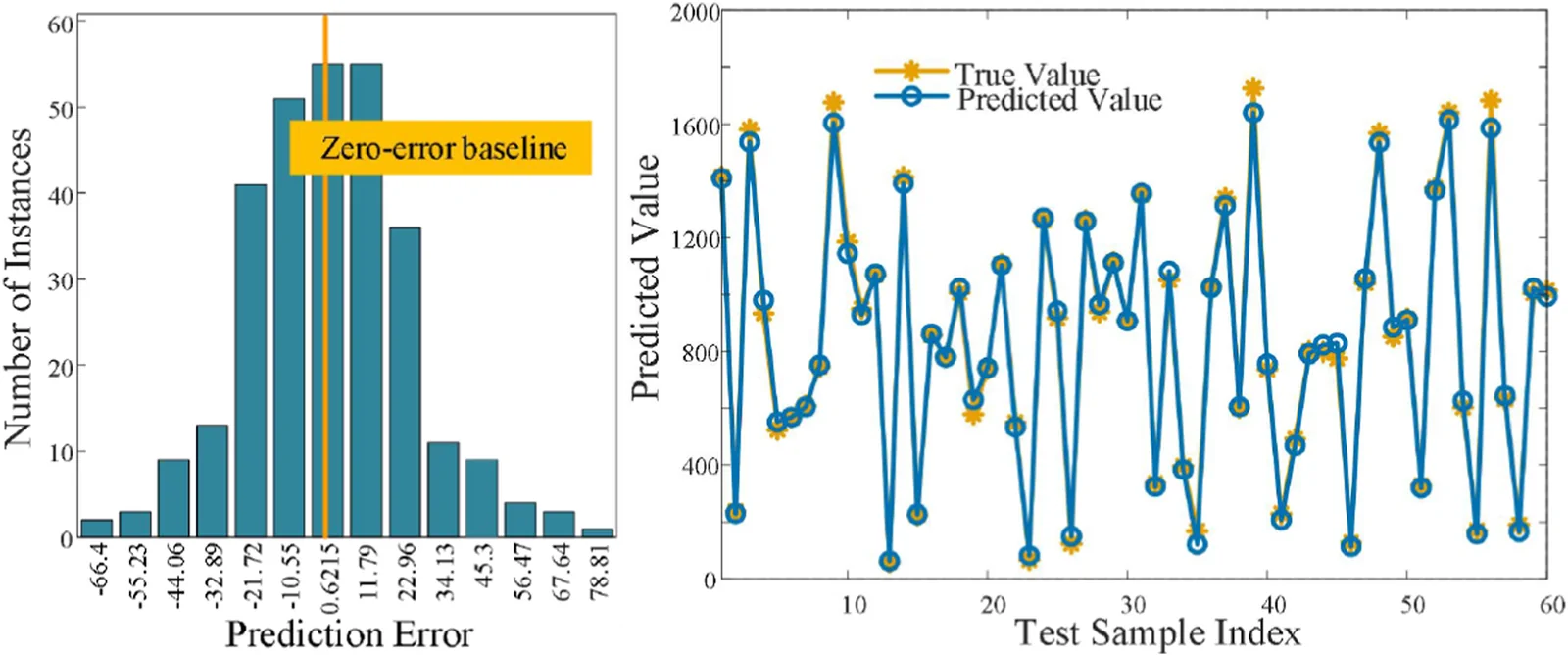
Error histogram with 14 bins and a comparison of test set prediction results for a portion of the data.

**FIGURE 7:**
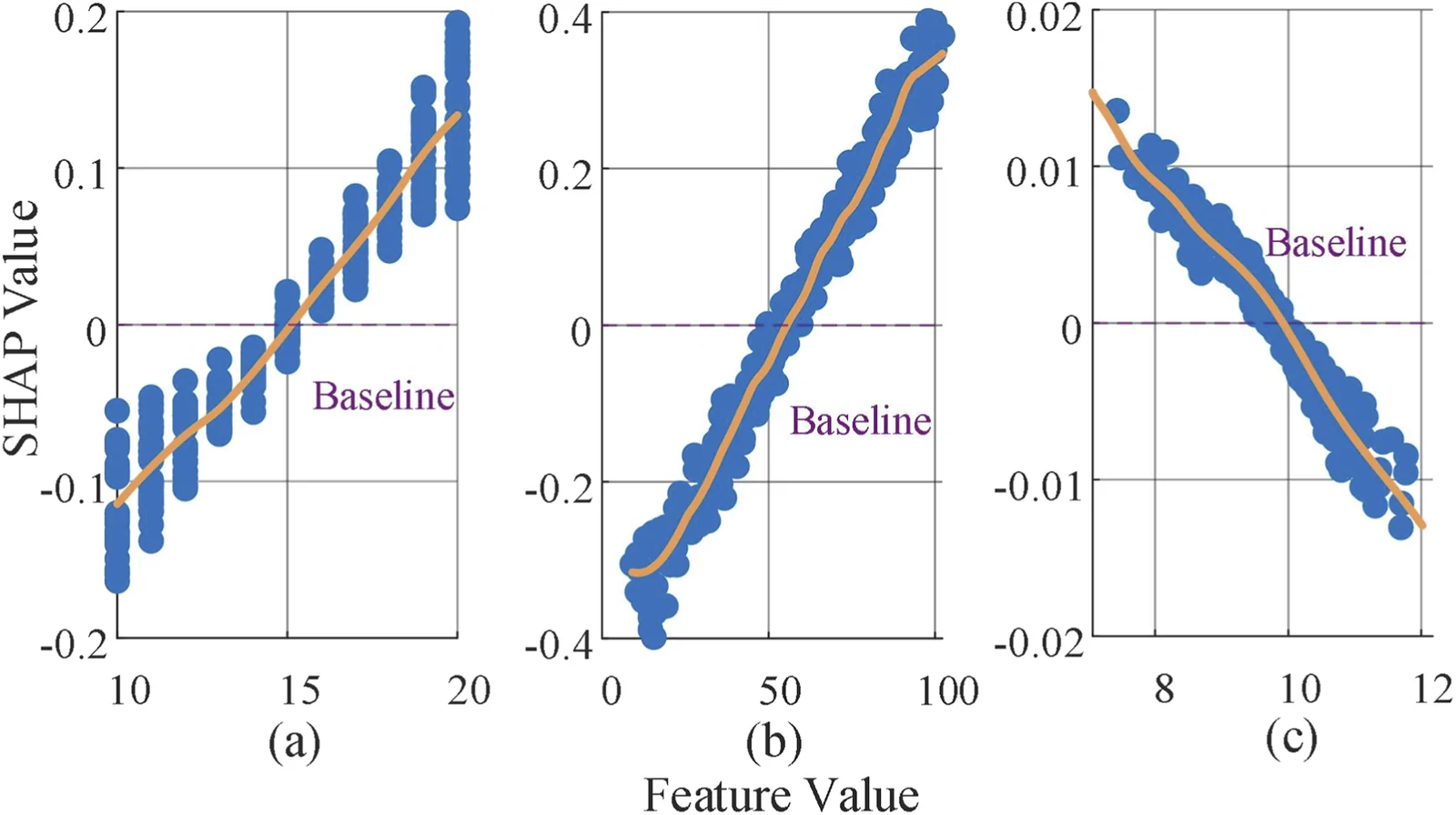
SHAP dependency plots for the top three features. **(a)** active tasks **(b)** the number of available robots **(c)** spatial distances between tasks and robots.

Additionally, we conducted a rigorous comparative analysis of 11 different algorithms to identify the optimal prediction model. These include classical shallow learning methods (KELM, ELM), standard deep learning models (LSTM, GRU, CNN-LSTM-Attention (Zhao et al., 2024), and various hybrid meta-heuristic optimized networks (GA-BP, PSO-DNN, NGO-LSSVM (Liu et al., 2024), DBO-ELM (Zhang et al., 2024a), COA-ESN (Xing et al., 2025). The performance metrics include $R^{2}$ , mean absolute error (MAE), mean squared error (MSE), and mean absolute percentage error (MAPE), for both training and testing phases are detailed in Table 2.

**TABLE 2:** Comparative analysis of predictive performance across different deep prior models.

Model	*R* ^2^		MAE		MSE		MAPE	
	Train	Test	Train	Test	Train	Test	Train	Test
SABO-LSTM	0.9973	0.9967	17.62	18.70	492.78	565.75	0.0459	0.0416
KELM	0.9976	0.9965	16.10	19.09	430.09	614.41	0.0361	0.0408
NGO-LSSVM	0.9971	0.9964	18.08	19.68	537.42	608.75	0.0469	0.0526
GRU	0.9965	0.9966	19.21	19.36	616.94	639.33	0.0478	0.0473
ELM	0.9968	0.9962	18.62	21.16	550.35	779.30	0.0477	0.0634
DBO-ELM	0.9966	0.9960	19.21	21.29	590.88	737.02	0.0525	0.0528
LSTM	0.9944	0.9941	24.75	25.65	992.36	1130.05	0.0724	0.0753
CNN-LSTM-ATTENTION	0.9947	0.9934	23.94	26.18	960.17	1158.61	0.0642	0.0734
GA-BP	0.9921	0.9909	28.81	33.08	1382.45	1768.06	0.0864	0.1037
PSO-DNN	0.9894	0.9880	33.73	39.79	1799.79	2447.70	0.1126	0.1264
COA-ESN	0.9801	0.9805	45.75	46.91	3526.81	3759.77	0.1314	0.1354

As evidenced by the experimental results, the proposed SABO-LSTM demonstrates superior performance across the most critical metrics among all tested models. SABO-LSTM achieved the highest $R^{2}$ value of 0.9967 on the testing set. This indicates that the model can explain 99.67% of the variance in the optimal cost boundaries, outperforming standard LSTM 0.99406 and other optimized variants like PSO-DNN 0.98802. In terms of stability and precision, SABO-LSTM achieved the lowest MSE (565.7532) and MAE (18.6959) on the test set. Compared to the standard LSTM (MSE = 1130.0539), the SABO-optimized model reduced the prediction error by approximately 49.9%. Furthermore, compared to other hybrid models like the MSE of GA-BP and PSO-DNN, SABO-LSTM demonstrates an improvement in order-of-magnitude, validating the efficacy of the Subtraction-Average-Based Optimizer in navigating the hyperparameter space. The SABO-LSTM model shows only a minimal gap between its training and test metrics. In contrast, models such as ELM and PSO-DNN exhibit a significant gap, with test errors much higher than training errors, which show a sign of overfitting. Accordingly, Table 2 is used as the primary and unified evaluation of the predictive performance of the SABO-LSTM model, while larger-scale datasets are reserved for the subsequent system-level scalability evaluation rather than for an additional standalone prediction-error comparison.

### 5.3 Comparative analysis of optimisation algorithm experiments

To evaluate the effectiveness of the proposed Guided Whale Optimization Algorithm (GWOA), we compare it against six representative metaheuristic algorithms: the standard Whale Optimization Algorithm (WOA), Particle Swarm Optimization (PSO), Group-Search-based WOA (GSWOA) (Hu et al., 2024), Learning-Enhanced Evolution (LEE) (Zhang et al., 2024b), Cooperative Coevolution Optimization (CCO) (Qi et al., 2023), the Local Greedy Construction (LGC) (Ding et al., 2023) heuristic, and the Wave Optics Optimizer (WOO) (Peng et al., 2026). All algorithms were tested under identical conditions with fixed task sets and resource constraints, with the objective of minimizing the composite execution cost. The population size for all algorithms is set to N=30 , with a maximum iteration count of 500. It should be noted that this comparative experiment evaluates the standalone optimization performance of the whale-based algorithms. The SABO-LSTM prior-guidance module is not introduced in this setting, so the comparison focuses on the intrinsic search capability of the optimizer itself.

As shown in Figure 8, GWOA exhibits a noticeably lower initial fitness value compared with WOA, PSO, and CCO. Since the SABO-LSTM prior-guidance module is disabled in this experiment, this initial advantage is primarily attributed to the chaotic initialization strategy, which improves the diversity and distribution of the initial population and increases the likelihood of sampling promising regions of the search space.

**FIGURE 8:**
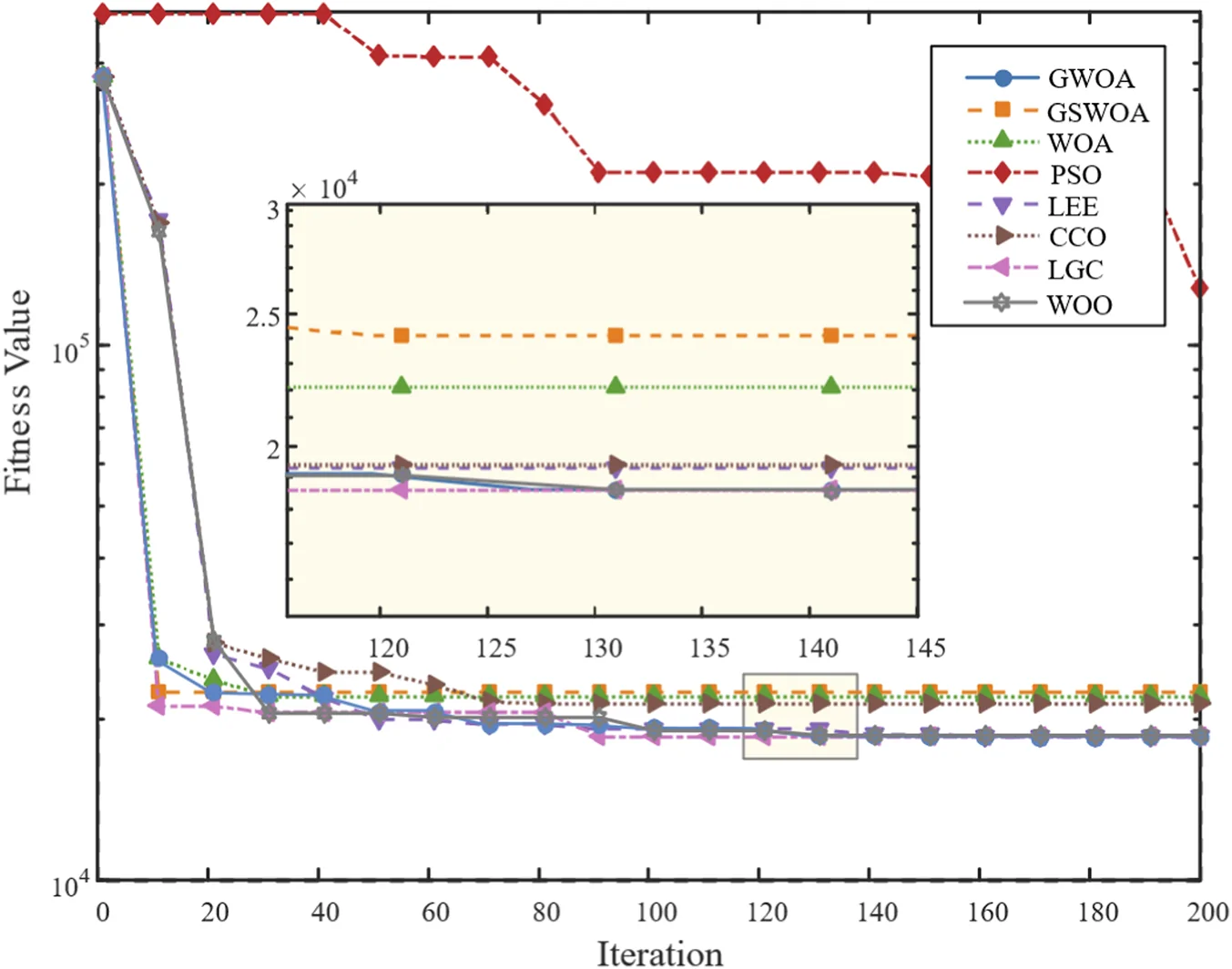
Iterative mapping of minimal-cost allocation by eight algorithms under defined conditions.

Additionally, PSO shows clear stagnation after approximately 40 iterations, with its fitness curve flattening at a relatively high cost level, indicating premature convergence. Similarly, WOA and GSWOA converge early but remain trapped in suboptimal regions. LEE and CCO demonstrate moderate improvements but suffer from oscillations and slower refinement in later iterations. WOO shows a relatively smooth convergence trajectory, indicating that its search dynamics are more effective in avoiding severe oscillations. However, its convergence curve still stabilizes at a fitness level slightly above that of GWOA, suggesting that WOO improves search stability but does not fully resolve the loss of global optimality under the discrete allocation constraints considered in this work. By contrast, GWOA maintains a steady downward trend throughout the optimization process. This behavior can be attributed to the target-guided adaptive control of the parameter (a) and the gap-aware Levy flight perturbation, which together enhance exploration when the solution is far from the predicted optimum and gradually shift toward exploitation as convergence progresses.

As shown in Figure 9, GWOA achieves the lowest median fitness with a tightly concentrated distribution, indicating strong robustness and repeatability. In comparison, GSWOA and LEE exhibit higher medians and broader spreads, reflecting sensitivity to initialization. CCO shows substantial dispersion, suggesting unstable convergence behavior under discrete constraints. WOO also presents a relatively compact distribution, with most fitness values concentrated near the lower-cost region, which confirms its good robustness across repeated runs. Compared with GSWOA, LEE, and CCO, WOO produces superior solutions, though the difference is not significant. The right panel provides a direct comparison between GWOA and standard WOA. The median fitness of GWOA is approximately $1.86\times 10^{4}$ , significantly lower than that of WOA. This improvement validates the effectiveness of the guided mechanisms—namely target-aware parameter control, Lévy-enhanced exploration, and probabilistic discretization—in addressing the limitations of standard WOA in high-dimensional binary allocation problems.

**FIGURE 9:**
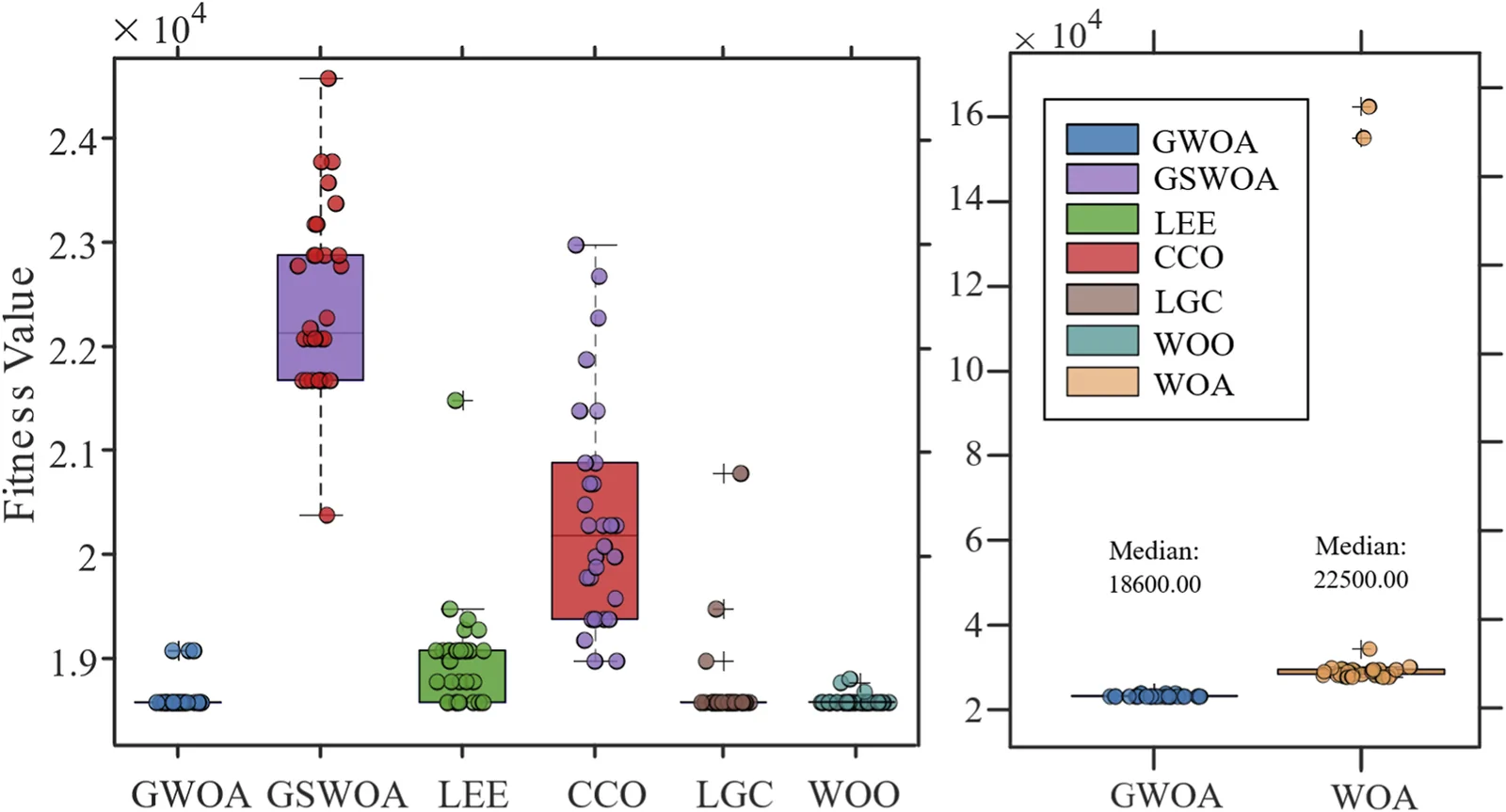
Performance comparison of optimization algorithms based on solution fitness distributions.

Across the repeated runs, GWOA achieved lower objective values and competitive convergence behavior than the compared metaheuristic methods under the considered experimental settings.

### 5.4 Comparison with strong MRTA/replanning baselines

While the previous subsection evaluates the proposed method against representative meta-heuristic optimizers, such comparisons alone are insufficient to fully characterize its performance within the broader landscape of multi-robot task allocation (MRTA) and dynamic replanning.

To provide a more comprehensive and rigorous evaluation, we further benchmark the proposed framework against several strong and widely adopted MRTA paradigms, including rolling-horizon exact optimization methods, namely rolling min-cost flow (R-MCF) and rolling assignment (R-Assign), a combinatorial optimization solver based on OR-Tools routing (ORT), a decentralized market-based method, i.e., auction-based MRTA (AuctionMRTA), and a contingency-aware replanning approach, namely the contingency-aware planner (CAP). In this comparison with MRTA/replanning baselines, GWOA is an allocation optimizer that does not incorporate the SABO-LSTM prior-guidance module. Therefore, the experiment assesses the task-allocation capability of the optimizer rather than the complete SABO-LSTM-guided planning framework. Table 3 summarizes the comparative results in terms of solution cost and computational efficiency.

**TABLE 3:** Performance comparison with stronger MRTA/replanning baselines.

Method	Best cost	Mean cost	Mean runtime (s)
GWOA	**18,600**	18,666.7	0.0305
R-MCF	24,310	24,310	0.1349
R-assign	24,310	24,310	0.1241
ORT	24,310	24,310	0.5856
CAP	18,600	18,800	**0.00159**
AuctionMRTA	**18,600**	**18,600**	0.0969

Bold values indicate the best value(s) within each performance metric, including best cost, mean cost, and mean runtime.

The above results highlight a fundamental trade-off in MRTA/replanning methods: exact optimization ensures optimality but struggles with adaptability, decentralized methods offer efficiency but lack global coordination, and reactive planners provide speed at the expense of solution quality. The proposed framework effectively bridges these gaps by combining learned priors with guided global optimization. It inherits the adaptability required for dynamic environments while preserving strong optimization capability, leading to consistently competitive performance across all evaluated baselines. These findings provide strong evidence that the proposed method is not only competitive with traditional meta-heuristics, but also compares favorably with state-of-the-art MRTA and replanning approaches across multiple methodological paradigms.

### 5.5 Ablation study and complexity-benefit analysis of GWOA

Although the previous subsection demonstrates that GWOA outperforms several representative optimization baselines, such aggregate performance gains alone do not fully clarify whether the proposed method derives its effectiveness from a principled guided design or merely from stacking multiple heuristic enhancements. To address this concern, we further conduct a component-wise ablation study and a complexity-benefit analysis of GWOA. The goal is to both quantify the contribution of each introduced module and to examine whether the additional computational overhead is justified by the observed performance gains. Table 4 reports the ablation results obtained by removing one component at a time from the full GWOA framework, while keeping all other settings unchanged. The ablation results are averaged over 30 independent runs under identical initialization settings.

**TABLE 4:** Ablation Study Results of GWOA (each column corresponds to one ablated variant).

Component	C1	C2	C3	C4	C5	C6	Effect size	P-value
Chaotic mapping	×	✓	✓	✓	✓	✓	0.4810	0.0084
Dynamic probability	✓	×	✓	✓	✓	✓	0.4240	0.0202
Levy flight	✓	✓	×	✓	✓	✓	0.8732	0.0009
Probabilistic rounding	✓	✓	✓	×	✓	✓	0.2159	0.2370
Diversity maintenance	✓	✓	✓	✓	×	✓	0.2727	0.1352
Opposition learning	✓	✓	✓	✓	✓	×	0.4399	0.0160
Relative loss (%)	19.75	30.05	47.56	4.20	15.95	35.85	-	-

The relative loss $R_{\mathrm{loss}}$ is computed based on the mean fitness over multiple runs, as defined in Equation 22.

$$R_{\mathrm{loss}}=\frac{{\bar{J}}_{r}-{\bar{J}}_{f}}{{\bar{J}}_{f}}\times 100\%,$$ (22)

where ${\bar{J}}_{r}$ denotes the average objective value after removing a given component, while ${\bar{J}}_{f}$ represents the average value of the full GWOA model. A positive relative loss directly reflects performance degradation caused by component removal, with larger values indicating higher component importance. In addition, statistical significance is evaluated using a two-tailed Wilcoxon signed-rank test with Holm-Bonferroni correction for multiple comparisons.

The effect size is quantified using the Wilcoxon effect-size measure $r=|Z|/\sqrt{N}$ , where Z is the standardized test statistic obtained from the Wilcoxon signed-rank test and N denotes the number of valid paired observations. This measure complements the p -value by reflecting the practical magnitude of the performance difference between the full GWOA and each ablated variant.

The ablation study in Table 4 confirms that the proposed GWOA is not a simple accumulation of heuristic tricks. The components contribute unequally: Lévy flight, dynamic probability control, opposition learning, and chaotic mapping all exhibit statistically significant impacts (p<0.05) . In contrast, probabilistic rounding and diversity maintenance show weaker effects, with p-values of 0.2370 and 0.1352, respectively, indicating non-significant contributions. These results are consistent with their corresponding effect sizes and relative losses (4.20% and 15.95%).

The complexity analysis indicates that the additional modules in GWOA do not alter the asymptotic complexity of the baseline WOA. All introduced operations (chaotic mapping, dynamic probability control, Lévy flight, opposition learning, probabilistic rounding, and diversity maintenance) add only moderate constant-factor overhead, such as one-time initialization, scalar updates, or extra candidate evaluations per iteration. In return, the ablation study reveals substantial performance gains: removing Lévy flight, opposition learning, or dynamic probability control causes relative losses of 47.56%, 35.85%, and 30.05%, respectively. Thus, the enhancements achieve a favorable complexity–benefit trade-off.

To further ensure statistical rigor, a Holm-Bonferroni correction is applied to control the family-wise error rate in the ablation study. Let m denote the number of hypotheses and $p_{\left(i\right)}$ represent the ordered p-values such that $p_{\left(1\right)}\leq \cdots \leq p_{\left(m\right)}$ . The Holm procedure sequentially tests each hypothesis using the adaptive threshold α/(m−i+1) , where α=0.05 .

Within the present ablation analysis, m=6 , corresponding to six ablated components. After applying the Holm-adjusted thresholds, Lévy flight remains highly significant, while chaotic mapping and opposition learning also remain significant under the corrected criteria. In contrast, dynamic probability control, diversity maintenance, and probabilistic rounding do not pass the corrected significance level, consistent with their relatively smaller effect sizes.

To further investigate whether these improvements in prediction accuracy translate into end-to-end optimization benefits, we conduct an ablation study under a unified multi-agent task allocation framework. The experimental environment is kept identical across all variants to ensure a fair comparison. A fixed task-agent configuration is used with (M = 150) agents and (N = 150) tasks. The cost matrix, resource constraints, and stochastic components are generated using a fixed random seed. The proposed guided GWOA framework is evaluated under four configurations: GWOA without LSTM prior, GWOA with standard LSTM prior, GWOA with SABO-LSTM prior, and GWOA with random pruning. All methods use identical hyperparameters, including a population size of 20 and a maximum iteration budget of 200, with fixed-iteration termination.

Table 5 reports the end-to-end comparison results. The proposed SABO-LSTM prior achieves the best overall optimization quality, obtaining the lowest final fitness value (6854.12), while maintaining competitive runtime (0.0778 s) . Compared with the standard LSTM prior, SABO-LSTM yields a consistent improvement in solution quality, indicating that improved prior accuracy is effectively translated into better downstream optimization performance.

**TABLE 5:** End-to-End impact of prior-guided mechanisms on runtime and optimization quality.

Method	Runtime (s)	Final fitness
GWOA with random pruning	**0.074317**	6951.5948
GWOA without LSTM prior	0.087266	6872.7559
GWOA with standard LSTM prior	0.091851	6937.7708
GWOA with SABO-LSTM prior	**0.077828**	**6854.1213**

Bold values indicate relatively better runtime performance (< 0.08 s) and the best Final fitness value.

### 5.6 Auxiliary optimizer baseline validation against MILP

To provide additional empirical grounding for the optimization layer, we conducted an auxiliary static VRPTW benchmark using the Solomon C101-15 instance. This instance contains one depot and 15 customers with spatial coordinates, demands, service costs, and strict time-window constraints. The vehicle capacity was set to 200, and the maximum number of vehicles was limited to $K=\min\left(8,N_{v}\right)$ , where $N_{v}$ denotes the available vehicle number. It should be emphasized that this experiment is not intended to evaluate the complete SABO-LSTM-guided contingency-aware replanning framework, because static benchmark instances do not provide scenario-consistent historical state-transition data required by the deep prior predictor. Instead, this experiment isolates the GWOA component and evaluates its optimizer-level computational behavior against an exact solver.

Specifically, an exact MILP model was first solved using MATLAB with the cut generation, heuristic search, and integer preprocessing. The resulting MILP objective was used as the exact reference. The same instance was then solved by GWOA with 30 search agents, 150 iterations, and five independent runs. During the GWOA runs, the MILP objective was used only as the target-cost reference for the guided search mechanism, while runtime, total distance, vehicle usage, and best and average performance were recorded.

As shown in Table 6, the exact MILP solver reached the optimal solution with an objective value of 142.1433 using 2 vehicles, but required 102.4508 s. By contrast, GWOA completed the optimization within 0.1880 s on average over five repeated runs, with the best objective value of 326.9194 and an average objective value of 359.9864. These results reveal a clear efficiency-quality trade-off. Although GWOA does not achieve the exact optimum under this static deterministic objective, it provides a substantial computational speed advantage.

**TABLE 6:** Auxiliary comparison results between exact MILP and GWOA.

Method	Run	Runtime (s)	Vehicles	Distance
MILP	-	102.4508	2	142.1433
GWOA	1	0.2156	4	354.7353
	2	0.1853	5	402.6760
	3	0.1786	4	356.2689
	4	0.1719	3	359.3323
	5	0.1889	4	326.9194
GWOA (best)		0.1719	3	326.9194
GWOA (average)		0.1880	5.80	359.9864

This outcome is consistent with the intended role of the proposed framework. The goal of GWOA in this work is not to replace exact solvers for static benchmark optimization, but to support rapid online replanning when dynamic contingencies trigger time-sensitive reallocation. Therefore, this auxiliary benchmark provides complementary optimizer-level evidence for the real-time suitability of the proposed planning framework.

### 5.7 Performance comparison and sensitivity analysis

As shown in Table 7, sensitivity analysis identifies learning rate ( η ) as the most influential hyperparameter, with optimal performance near 0.007–0.008, L2 regularization ( $\lambda_{L2}$ ) shows moderate sensitivity, while hidden dimension ( $N_{h}$ ) has negligible impact. Given its computational demands, SABO-LSTM is best suited for offline training scenarios, whereas hybrid approaches such as GWOA could be considered for real-time applications.

**TABLE 7:** Sensitivity analysis of key hyperparameters.

Parameter	Value	RMSE	$R^{2}$
η	0.00345	146.65	0.99883
	0.00552	141.09	0.99892
	0.00690	132.84	0.99904
	0.00828	124.45	0.99916
	0.01000	143.99	0.99887
$N_{h}$	59	140.73	0.99892
	69	150.59	0.99877
	79	143.91	0.99887
	89	156.68	0.99867
	99	150.86	0.99876
$\lambda_{L2}$	$3.32\times 10^{-6}$	131.30	0.99906
	$8.30\times 10^{-6}$	145.47	0.99885
	$1.66\times 10^{-5}$	135.68	0.99900
	$3.32\times 10^{-5}$	130.47	0.99908
	$8.30\times 10^{-5}$	161.13	0.99859

To further evaluate the proposed predictor, we introduced Att-DRL and GNN-PPO as additional modern learning-based baselines. These models represent two commonly used learning paradigms in MRTA-related studies, namely policy-learning-based sequential decision making and graph-structured representation learning. The comparison was conducted under the same prediction-oriented evaluation protocol using RMSE, MAE, MAPE, $R^{2}$ , and computational time, so as to assess their suitability for continuous deep-prior prediction.

As shown in Table 8, Att-DRL demonstrates limited suitability for this supervised regression task. This indicates that a pure reinforcement-learning formulation is ill-aligned with continuous prior estimation, primarily because it relies on reward-driven policy learning rather than direct regression-error minimization. Conversely, GNN-PPO achieves significantly stronger predictive performance, suggesting that graph-based representation learning effectively captures relational information in MRTA-related data. While GNN-PPO provides a strong learning-based benchmark, SABO-LSTM ultimately offers a more consistent and framework-compatible predictor for deep-prior generation. It successfully combines high explanatory accuracy, enhanced stability, and direct alignment with the supervised prediction objective of the proposed planning framework.

**TABLE 8:** Performance of additional learning-based baselines for prediction-module comparison.

Model	RMSE	MAE	MAPE	$R^{2}$	Time (s)
Att-DRL	507.25	436.74	1.65	0.38	572.15
GNN-PPO	44.35	32.29	0.11	0.99	127.40

### 5.8 Experimental validation of dynamic responses to different scenarios

To evaluate system resilience under stochastic transitions, we subjected the proposed framework to the six contingency scenarios defined in the problem formulation. The simulations covered all major perturbation types, including task set contraction (Scenarios 1–2), constraint intensification (Scenario 3), demand escalation (Scenarios 4–5), and resource volatility (Scenario 6). The quantitative response to these dynamic events is detailed in Table 9. To clarify the configuration of each compared variant, SABO-LSTM-WOA denotes a model in which the SABO-LSTM module provides deep-prior guidance to the standard WOA, while the GWOA-specific improvements, including target-gap adaptive control and dimension-wise opposition learning, are not introduced.

**TABLE 9:** Comparison of the redistribution processing time and results for the SABO-LSTM-WOA and SABO-LSTM-GWOA models across six scenarios.

Scenario	Occurrence time/min	Case change	SABO-LSTM-WOA		
			Taskc	Time/s	Result
Scenario 1	T+5	16×(9→7)	4	0.1010 ± 0.0037	323.92 ± 15.47
Scenario 2	T+10	12×(8→4)	4	0.1362 ± 0.0041	266.81 ± 5.48
Scenario 3	T+5	12×7	3	0.1514 ± 0.0062	556.51 ± 11.24
Scenario 4	T+10	13×(10→12)	10	0.1741 ± 0.0041	1062.2 ± 12.9
Scenario 5	T+5	15×(10→15)	9	0.1830 ± 0.0048	1361.9 ± 25.4
Scenario 6	T+10	(14→9)×9	5	0.1582 ± 0.0039	1267.6 ± 12.6

Scenario	Occurrence time/min	Case change	SABO-LSTM-GWOA		
			Taskc	Time/s	Result
Scenario 1	T+5	16×(9→7)	3	0.0983 ± 0.0017	319.59 ± 10.31
Scenario 2	T+10	12×(8→4)	2	0.1324 ± 0.0027	265.81 ± 3.33
Scenario 3	T+5	12×7	1	0.1503 ± 0.0043	552.59 ± 12.26
Scenario 4	T+10	13×(10→12)	8	0.1732 ± 0.0032	1041.6 ± 10.2
Scenario 5	T+5	15×(10→15)	6	0.1820 ± 0.0031	1290.0 ± 14.8
Scenario 6	T+10	(14→9)×9	3	0.1559 ± 0.0021	1251.2 ± 10.6

For the six contingency-scenario experiments, each algorithmic configuration was independently executed 50 times under identical problem settings. The objective value and computational runtime are reported as mean ± standard deviation over these 30 runs to account for the stochasticity of the metaheuristic optimization process.

Comparative analysis reveals that the SABO-LSTM-GWOA model consistently outperforms the SABO-LSTM-WOA baseline across all metrics. Specifically, the GWOA variant demonstrates superior stability, evidenced by reduced task reassignment counts and lower objective function values, while maintaining sub-second computational latency even under high-stress conditions. This underscores the model’s robustness in generating rapid, high-quality reallocations amidst abrupt dimensional shifts or agent failures. Although the LSTM predictor is trained offline, the proposed framework maintains robustness to out-of-distribution events because the neural model only provides probabilistic priors rather than final decisions. The downstream optimization layer can still explore feasible solutions even when the prediction deviates from unseen scenarios. This hybrid design improves generalization compared with purely learning-based allocation policies.

### 5.9 Scalability analysis under high-dimensional constraints

Having established the system’s rapid response capability in standard settings, we extended the evaluation to assess scalability under high-dimensional constraints. We conducted medium- and large-scale stress tests to verify whether the model maintains its superiority as the problem size and state space increases. For this analysis, we generated synthetic instances at two scales: medium ( 100−200 agents/tasks) and large ( 500−1000 agents/tasks). To ensure convergence stability and accelerate the search process in these expanded search spaces, the initial seed computations were pre-trained on datasets within these respective ranges.

The comparative results across the six contingency scenarios are detailed in Table 10. Model 1 is the SABO-LSTM-WOA, while Model 2 is the SABO-LSTM-GWOA. Even with the dramatic increase in state space dimensions, Model 2 consistently achieves lower objective function values compared to Model 1 baseline, while maintaining a marginal advantage in computational time. Notably, in large-scale scenarios, the proposed method resolves the reallocation problem within 15.3 s. Despite the increase in problem scale, the computational latency remains on the order of seconds, which is still acceptable for practical deployment. The experimental validation is restricted to dynamic multi-robot task-allocation and contingency-replanning settings that are consistent with the mathematical formulation considered in this work, rather than extending the evaluation to routing benchmarks requiring substantially different decision structures.

**TABLE 10:** Model comparison validation experiments under medium-scale and large-scale conditions across six scenarios.

Scenario	Case change	Model 1		Model 2	
		Time/s	Result	Time/s	Result
1	100×(190→195)	1.465 ± 0.301	13,349 ± 94	1.460 ± 0.028	13,212 ± 78
	757×(868→858)	14.683 ± 0.146	21,714 ± 236	14.480 ± 0.106	21,287 ± 172
2	172×(150→140)	2.132 ± 0.015	9215 ± 101	2.130 ± 0.806	9005 ± 91
	529×(618→598)	9.119 ± 0.087	36,045 ± 213	9.026 ± 0.058	36,026 ± 118
3	152×108	1.778 ± 0.010	1082 ± 105	1.752 ± 0.007	990 ± 77
	729×989	14.468 ± 0.171	90,137 ± 226	14.447 ± 0.148	89,811 ± 191
4	162×(199→204)	2.236 ± 0.012	1722 ± 104	2.224 ± 0.011	1507 ± 85
	576×(521→531)	8.826 ± 0.247	8247 ± 214	8.808 ± 0.132	8174 ± 153
5	171×(183→193)	2.234 ± 0.016	13,913 ± 121	2.232 ± 0.015	13,565 ± 100
	796×(809→829)	15.511 ± 0.127	24,506 ± 153	14.162 ± 0.113	24,043 ± 129
6	(119→114)×198	1.772 ± 0.014	10,180 ± 76	1.747 ± 0.011	10,024 ± 54
	(796→786)×815	15.699 ± 0.130	32,469 ± 193	15.288 ± 0.107	32,437 ± 168

## 6 Conclusion

In this paper, we proposed a generative real-time mission planning framework for multi-robot contingencies in stochastic environments. Our framework introduces a deep prior-guided mechanism that uses a SABO-optimized LSTM network to extract latent spatiotemporal features and map discrete state transitions to continuous cost intervals, thereby compressing the search space. For precise plan reallocation, we enhanced the Whale Optimization Algorithm with target-gap adaptive control and dimension-wise opposition learning, which better balances global exploration and local exploitation. Compared to traditional periodic reallocation methods, our framework generalizes effectively to a broader range of simulation scenarios while maintaining both flexibility and computational efficiency. The primary contribution of this work is an engineering-oriented generative planning framework for real-time multi-robot contingency management. The secondary contribution is a set of practical enhancements to the whale optimization algorithm to improve convergence efficiency under dynamic constraints. The experimental results validate that the model achieves sub-second responsiveness across six specific contingency scenarios and preserves seconds-level scalability in large-scale stress tests. Future work will focus on developing a decentralized decision-making mechanism for bandwidth-limited settings and incorporating the coupling effects of heterogeneous physical constraints.

## Data Availability

The original contributions presented in the study are included in the article/supplementary material, further inquiries can be directed to the corresponding author.

## Nomenclature

U: Set of available agents/robots
$u_{i}$: The i -th agent
M: Number of agents
$A_{i}=\left(p_{i},c_{i},v_{i}\right)$: Attribute tuple of agent $u_{i}$ (m), including position, capability vector, and velocity (m/s)
$p_{i}$: Current position of agent $u_{i}$ (m)
$c_{i}$: Capability/resource vector of agent $u_{i}$ (kg)
$v_{i}$: Maximum velocity of agent $u_{i}$ (m/s)
O(t): Set of active task orders at time t
$o_{j}$: The j -th task/order
N: Number of active tasks
$l_{j}$: Target location of task $o_{j}$ (m)
$q_{j}$: Resource or capability requirement of task
$\left[t_{j}^{s},t_{j}^{e}\right]$: Time window of task $o_{j}$ (s)
$w_{j}$: Priority weight of task $o_{j}$
$\lambda_{j}$: Risk level of task $o_{j}$
X(t): Assignment decision matrix at time t
$x_{ij}$: Binary decision variable; $x_{ij}=1$ if agent $u_{i}$ is assigned to task $o_{j}$
$S_{t}=\left(U_{t},O_{t}\right)$: System state at time t
E: Set of contingency events
$e_{r}$: Risk-induced task termination
$e_{l}$: Relevance-loss event
$e_{c}$: Constraint-mutation event
$e_{n}$: Dependency-injection or new-task event
$e_{i}$: Criticality-escalation event
$e_{f}$: Agent-paralysis event
$X^{\prime }$: Regenerated assignment matrix after contingency handling
J: Overall objective function
$J_{e}$: Execution cost component
$J_{s}$: Stability penalty component
ω: Weighting factor of the stability penalty
$E_{ij}\left(v_{o}\right)$: Energy consumption of assigning agent $u_{i}$ to task $o_{j}$ under velocity profile $v_{o}$ (Wh)
$\Phi_{\Delta }\left(\tau_{ij}\right)$: Time-window deviation penalty (s)
$P_{s}^{ij}$: Success probability of agent executing task
$\beta_{1},\beta_{2},\beta_{3}$: Weighting coefficients for energy, punctuality, and reliability terms
$X_{p}$: Previous assignment plan before contingency occurrence
$O_{A}$: Set of affected tasks
Ω: Original combinatorial search space
$\Omega^{\prime }$: Pruned search space after prior-guided compression
$\Omega_{r}^{\prime }$: Search subspace after hard feasibility pruning
$\Omega_{p}^{\prime }$: Search subspace after prediction-guided filtering
$\Omega_{f}$: Search subspace satisfying physical feasibility constraints
${\hat{R}}_{min}$: Predicted lower bound of reliability
$J_{p}$: Prior-estimated risk-adjusted reference cost
$N_{p}$: Population size in SABO/GWOA
$T_{max}$: Maximum iteration number
η: Learning rate of the LSTM model
$N_{h}$: Number of hidden units in the LSTM model
$\lambda_{L2}$: L2 regularization coefficient
P: Deep prior vector generated by the SABO-LSTM predictor
